# Applying machine learning to balance performance and stability of high energy density materials

**DOI:** 10.1016/j.isci.2021.102240

**Published:** 2021-02-26

**Authors:** Xiaona Huang, Chongyang Li, Kaiyuan Tan, Yushi Wen, Feng Guo, Ming Li, Yongli Huang, Chang Q. Sun, Michael Gozin, Lei Zhang

**Affiliations:** 1 Institute of Chemical Materials, China Academy of EngineeringPhysics (CAEP), Mianyang, 621900, China; 2 CAEP Software Center for High Performance Numerical Simulation, Beijing, 100088, China; 3 Department of Mechanical Engineering, City University of Hong Kong, 83 Tat Chee Avenue, Kowloon, 999077, Hong Kong, China; 4 Key Laboratory of Low-dimensional Materials and Application Technology (Ministry of Education), School of Materials Science and Engineering, Xiangtan University, Xiangtan, 411105, China; 5 School of Physical Science and Information Technology, Liaocheng University, Liaocheng, 252000, China; 6 EBEAM, Yangtze Normal University, Chongqing, 408100, China; 7 NOVITAS, Nanyang Technological University, Singapore, 639798, Singapore; 8 School of Chemistry, Faculty of Exact Science, Tel Aviv University, Tel Aviv, 69978, Israel; 9 Tel Aviv University Center for Nanoscience and Nanotechnology, Tel Aviv, 69978, Israel; 10 Center of Advanced Combustion Science, Tel Aviv University, Tel Aviv, 69978, Israel; 11 Laboratory of Computational Physics, Institute of Applied Physics and Computational Mathematics, Beijing, 100088, China

**Keywords:** Computational Materials Science, Computational Method in Materials Science, Energy Materials, Materials Design

## Abstract

The long-standing performance-stability contradiction issue of high energy density materials (HEDMs) is of extremely complex and multi-parameter nature. Herein, machine learning was employed to handle 28 feature descriptors and 5 properties of detonation and stability of 153 HEDMs, wherein all 21,648 data used were obtained through high-throughput crystal-level quantum mechanics calculations on supercomputers. Among five models, namely, extreme gradient boosting regression tree (XGBoost), adaptive boosting, random forest, multi-layer perceptron, and kernel ridge regression, were respectively trained and evaluated by stratified sampling and 5-fold cross-validation method. Among them, XGBoost model produced the best scoring metrics in predicting the detonation velocity, detonation pressure, heat of explosion, decomposition temperature, and lattice energy of HEDMs, and XGBoost predictions agreed best with the 1,383 experimental data collected from massive literatures. Feature importance analysis was conducted to obtain data-driven insight into the causality of the performance-stability contradiction and delivered the optimal range of key features for more efficient rational design of advanced HEDMs.

## Highlights

• Crystal-level quantum mechanics calculations of 21,648 physicochemical data of 153 HEDMs
• Machine learning of detonation and stability of HEDMs and experimental validation
• Data-driven insight into the causality of performance-stability contradiction
• Optimal range of key features for rational design of advanced HEDMs

## Graphical abstract

**Figure:**
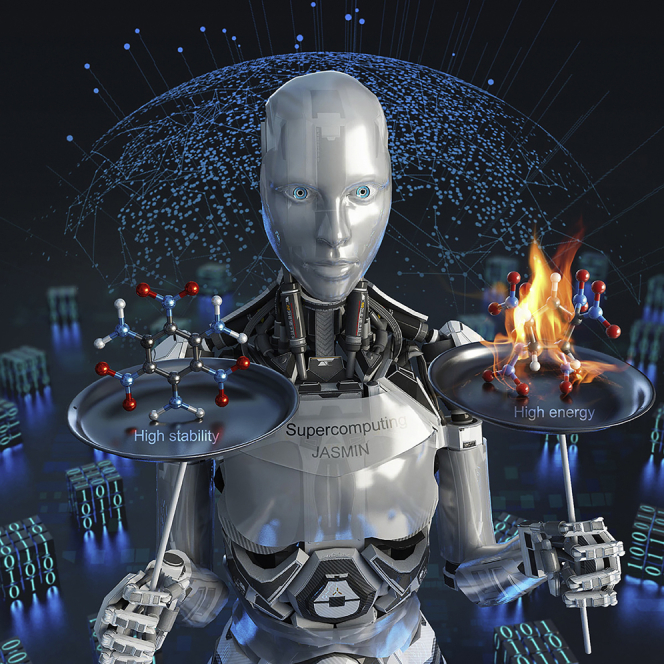

## Introduction

High energy density materials (HEDMs), also known as energetic materials, mainly refer to explosives, propellants, and pyrotechnics depending on their properties, formulations, and intended applications (Agrawal, 2010). As an alternative way to the use of human work, utilization of HEDMs alleviates arduous tasks of quarrying, mining, building tunnels, taming rivers, and building roads/rails by quick release of large amounts of gas through controlled chemical reactions, making laborious activities more efficient and economical, thereby playing an important role in accelerating the progress of human civilization (Klapötke, 2015). In this modern era, the dramatic increase in global population and concomitant depletion of available resources are demanding more advanced HEDMs to safely explore ultra-deep mineral deposits of earth, conduct space exploration, and so forth. Nonetheless, the development of HEDMs has been an incredibly slow process, with its milestones being the discovery of black powder at around 220 BC, the invention of trinitrotoluene (TNT) in the 1880s, the synthesis of widely used octahydro-1,3,5,7-tetranitro-1,3,5,7-tetrazocine (HMX) during World War II, and the development 2,4,6,8,10,12-hexanitro-2,4,6,8,10,12-hexaazatetracyclo-[5.5.0.0^3,11^.0^5,9^]-dodecane (CL-20) in 1980s. The currently required capability of HEDMs demands high detonation performance (maximum detonation velocity *D*, maximum detonation pressure *p*_*C-J*_, and maximum heat of explosion *Q*_*max*_) with simultaneous maximum stability, which includes minimal chemical degradation upon storage, no phase transition to a polymorph with an inferior performance, and no initiation upon accidental mechanical impact, friction, and non-mechanical *stimuli*, such as exposure to light, to irradiation in a non-visible spectral range, to electrostatic discharge, etc. Unfortunately, currently used state-of-the-art HEDMs have difficulty in addressing in a satisfactory manner all these conflicting criteria.

Possibly, the extremely high hazard involved and the high cost of the experimental research on HEDMs, as well as the long-term life cycle of their characterization, manufacturing, testing, and inspection, could be the excuses for the slow development of HEDMs (Wejsa, 2014). Furthermore, one more important reason for the slow development of HEDMs is the contradiction between their high detonation performance and high stability (Shukla et al., 2017; Sabin, 2014; Yu et al., 2020; Zhang et al., 2014; Tang et al., 2020; Jiao et al., 2018). A high detonation performance of HEDMs relies on the large energy difference between the reactants and the reaction products, whereas the high stability of HEDMs requires a sufficiently high energy barrier to prevent the uncontrolled initiation of such reactions (Jiao et al., 2018). Known molecular design strategies in reaching high detonation performance of HEDMs, in many perspectives, conflict with those in reaching high stability. For example, the presence of nitro group, N-oxide group, nitramino, furazan, furoxan, or oxadiazole in energetic molecules can increase the oxygen balance (OB) of HEDMs; and incorporation of azido group, N = C, and N = N bonds into energetic molecules can lead to the increase in the nitrogen content (ρ_N_) of HEDMs (Yu et al., 2020). Both strategies were capable of leading to the increase of the density (ρ) and heat of formation of the energetic molecules, facilitating the promotion of their detonation performance (Hu et al., 2020). However, when the increase in the OB leads to better performance, it pays the price of higher sensitivity and lower stability of the resulting HEDM (Wang et al., 2018; Li et al., 2020a, 2020b, 2020c). Moreover, the increase of ρ_N_ in various nitrogen-rich HEDMs inevitably introduces N–N bonds, the low bond strength of which would result in reduced stability of such molecules (Tang et al., 2017; Zong et al., 2020).

Besides the aforementioned factors, numerous crystal-level physicochemical parameters, such as intermolecular interactions, energetics of the compound, crystal packing arrangements, and ratio of component molecules in co-crystals, were also found to significantly influence the detonation performance and stability of HEDMs. For example, hydrogen bonding (HB) plays a vital role in optimizing both detonation performance and crystal stability. Crystal structures of HEDMs rich in HBs were shown to have better stability, and expressed enhanced heat resistance and improved insensitivity to impact and shock *stimuli*, versus compounds with limited HB amount in their crystal structures (Rupeng et al., 2019; Li et al., 2020a, 2020b, 2020c; Zhang et al., 2019a, 2019b, 2019c, 2019d). For example, when compared with neutral tetranitroamino HEDMs, corresponding energetic salts showed better thermal and mechanical stabilities, owing to the extensive HB interactions between cations and anions (Lang et al., 2020). However, unfortunately, high content of hydrogen in energetic molecules would in turn lead to decreased ρ and reduced detonation performance of HEDMs.

Over the past decade, the machine learning (ML) algorithms have emerged, rapidly developed, and been extensively used in materials research (Zahrt et al., 2019; Sanchez-Lengeling and Aspuru-Guzik, 2018; Lu et al., 2018; Zhong et al., 2020; Granda et al., 2018; Butler et al., 2018). Compared with the traditional physics-based or knowledge-driven approach, ML algorithms are data driven and have an important characteristic trait of easily handling very complex datasets, providing accurate predictions, and helping in deciphering new knowledge (Shi and Iyengar, 2020). Therefore, ML is an extremely promising technique to handle the detonation-stability contradiction conundrum and accelerate the progress in the design of advanced HEDMs.

Noteworthy, reasonable feature descriptors and suitable models are the key to the successful application of the ML tool in solving the detonation-stability contradiction. According to the literature, commonly employed feature descriptors of HEDMs are the structure and energy properties of individual molecules, rather than those of crystals, owing to the lack of a reliable quantum chemical approach to handle the periodic structures of crystals and the weak in-crystal intermolecular interactions. In general, the feature descriptors include counts of atoms, bonds, and groups; OB; nitrogen-to-carbon ratio; bond types; electro-topological state; fingerprinting; Coulomb matrices; highest occupied and lowest unoccupied molecular orbitals; nitrogen charges at the bond midpoint; the lowest negative charge on nitro group; molecular polarizability; ionization energy; etc. (Elton et al., 2018; Barnes et al., 2018; Wang et al., 2012; Zhang et al., 2017; Cho et al., 2005; Kang et al., 2020). A considerable part of these feature descriptors is quantum chemical calculation results for HEDM molecules. Till date, the models that have been designed, developed, and employed for ML of HEDMs include multiple linear regression, artificial neural network (ANN), kernel ridge regression (KRR), support vector regression, random forest (RF), *k*-nearest neighbors, decision tree, least absolute shrinkage, selection operator regression, Gaussian process regression, etc. (Xu et al., 2012; Wang et al., 2012; Fathollahi and Sajady, 2018; Elton et al., 2018; Barnes et al., 2018; Kang et al., 2020; Zhang et al., 2017; Chandrasekaran et al., 2019; Nefati et al., 1996). The validation metrics derived from these data-driven models brought a high confidence in their use for a reasonably reliable prediction of *D*, *p*_*C-J*_, *Q*_*max*_, heat of formation, impact sensitivity, decomposition temperature (*T*_*d*_), and other critical properties of HEDMs (Xu et al., 2012; Wang et al., 2012; Fathollahi and Sajady, 2018; Elton et al., 2018; Barnes et al., 2018, Barnes et al., 2020; Kang et al., 2020; Gupta et al., 2016; Chandrasekaran et al., 2019; Nefati et al., 1996).

In this study, we made an effort to bridge the gap that exists between the nature of discrete molecule and extended periodic solid material (Mancuso et al., 2020) by conducting quantum mechanics calculations on parameters of physicochemical properties of 153 HEDMs, directly at the crystal level, instead of more common calculations dealing with individual molecules in a gas phase. These 21,648 calculated data were then used as input for ML. Five models were, respectively, trained and evaluated in predicting the *D*, *p*_*C-J*_, *Q*_*max*_, *T*_*d*_, and lattice energy (*LE*) of HEDMs; 1,383 experimental data were collected from a comprehensive literature search to verify the reliability of our current calculations and ML predictions. Subsequently, feature importance analysis was carried out; the features were classified into contradictory and non-contradictory categories, and finally the optimal range of key features was recommended for use in a rational molecular design of novel advanced HEDMs.

## Results

### Materials informatics and machine learning

**Figure 1:**
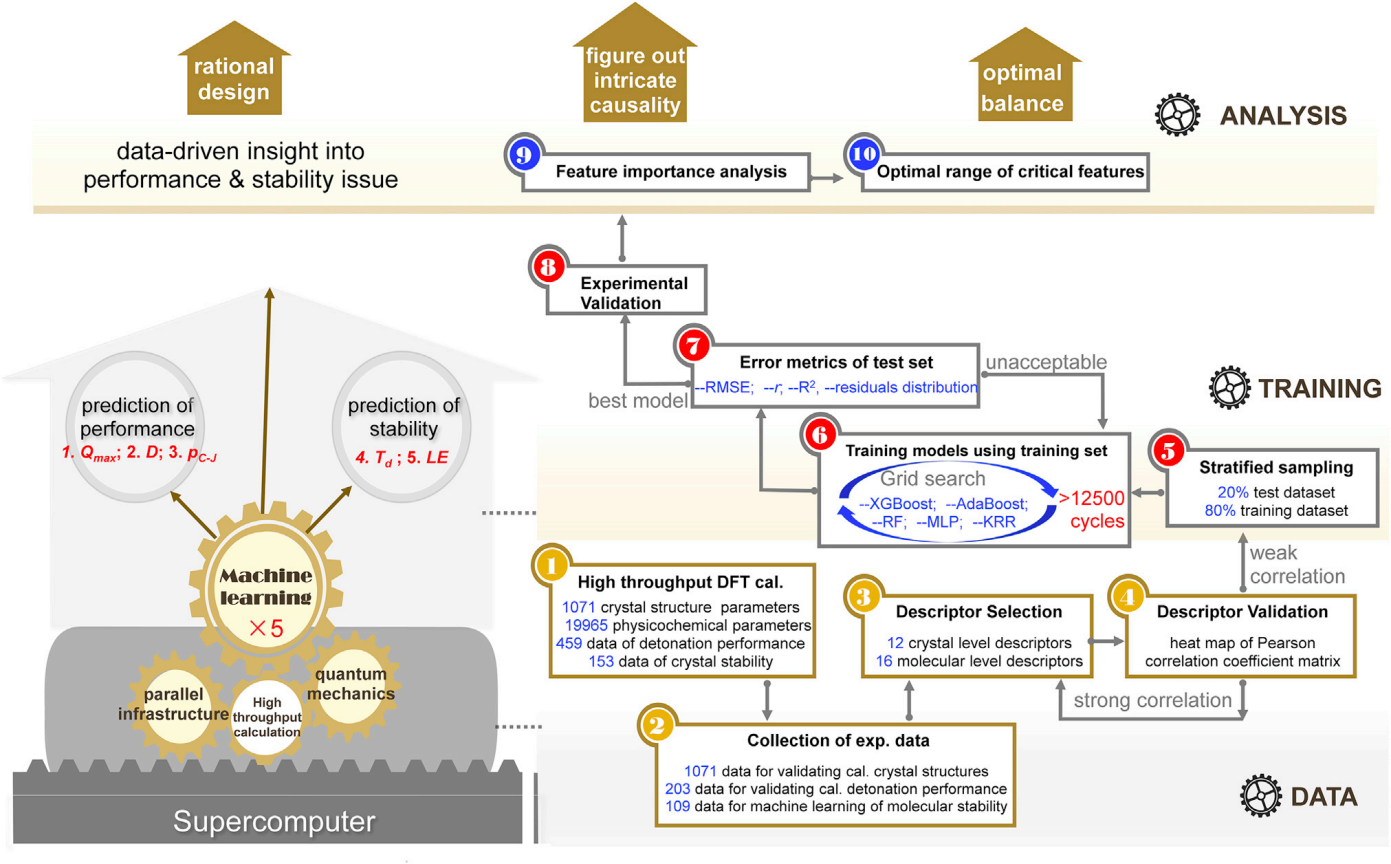
A flow chart of ML process for balancing detonation performance and stability of HEDMs High-throughput DFT calculations on supercomputers are the basis of data preparation for ML. Five ML models were trained and evaluated using stratified sampling and leave-one-out cross-validation, and the collected massive experimental data help to validate the predictions. Feature importance ranking provides a data-driven insight into the performance-stability contradiction and presents guidance on the optimal range of critical features.

**Figure 2:**
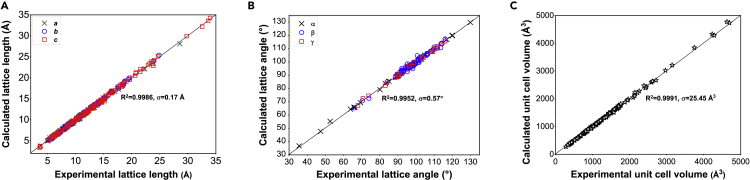
DFT calculation of 1,071 crystal structure parameters of HEDMs studied herein and their experimental validation (A–C) (A) Lattice constants (*a*, *b*, and *c*); (B) lattice angles (*α*, *β*, and *γ*); and (C) unit cell volumes of the 153 HEDMs studied.

Figure 1 shows a flow chart of the use of ML technique for addressing the contradiction between detonation performance and stability of HEDMs. Current study concentrates on 153 reported HEDMs, which are all stable at ambient conditions, and their single-crystal X-ray crystallography data are available. The physicochemical parameters, detonation performance parameters, and stability properties were calculated at the crystal level, by using a recently developed density functional theory (DFT) software, high accuracy atomistic simulation package for energetic materials (HASEM) (Zhang et al., 2016a, 2016b). J parallel adaptive structured mesh applications infrastructure (JASMIN) facilitates HASEM to adapt to modern supercomputers, thereby allowing high-throughput DFT calculations (Mo et al., 2010). The lattice parameters and atomic coordinates from the experimental data were set as the input of HASEM software to optimize structures. Minute discrepancies between the calculated and experimental results of 1,071 crystal structure parameters confirm the high reliability of HASEM method in describing the crystal structures of HEDMs (Figure 2).

#### Dataset

Based on these optimized structures, 19,965 physicochemical parameters were calculated and employed as feature descriptor candidates for ML of HEDMs (Tables 1 and S2). Furthermore, 459 calculated values of *D*, *p*_*C-J*_, and *Q*_*max*_; 153 calculated values of *LE*; and 109 collected experimental data of *T*_*d*_ were used as outcomes to train the ML models for predicting the detonation performance, crystal stability, and molecular stability of HEDMs, respectively. Additional 203 collected experimental data were used to validate the results of ML predictions (Table S3; supplemental information).

**Table 1:** Scoring metrics of the 5 ML models in predicting detonation and stability properties (*D*, *p*_*C-J*_, *Q*_*max*_, *T*_*d*_, and *LE*) of HEDMs, provided individually for the training set and test set

Properties		Models	Training dataset			Test dataset		
			RMSE	*r*	*R*^2^	RMSE	*R*	*R*^2^
Detonation performance	*Q*_*max*_	XGBoost	49.694	0.984	0.958	99.988	0.914	0.825
		AdaBoost	60.579	0.970	0.938	108.252	0.898	0.794
		RF	55.731	0.983	0.948	103.574	0.926	0.812
		MLP	78.539	0.947	0.896	101.070	0.909	0.821
		KRR	96.939	0.918	0.841	101.291	0.916	0.820
	***D***	**XGBoost**	**0.101**	**0.993**	**0.985**	**0.235**	**0.956**	**0.912**
		AdaBoost	0.114	0.991	0.981	0.274	0.942	0.879
		RF	0.143	0.986	0.970	0.276	0.943	0.878
		MLP	0.185	0.976	0.949	0.244	0.959	0.905
		KRR	0.256	0.950	0.903	0.291	0.941	0.864
	***p***_***C-J***_	**XGBoost**	**0.817**	**0.992**	**0.984**	**1.788**	**0.954**	**0.910**
		AdaBoost	1.084	0.987	0.972	2.426	0.920	0.835
		RF	1.097	0.987	0.971	2.360	0.924	0.843
		MLP	0.898	0.990	0.981	2.256	0.954	0.857
		KRR	1.983	0.951	0.905	2.812	0.902	0.778
Molecular stability	***T***_***d***_	**XGBoost**	**29.919**	**0.933**	**0.803**	**52.069**	**0.781**	**0.557**
		AdaBoost	26.932	0.947	0.840	54.347	0.741	0.518
		RF	21.229	0.970	0.901	54.153	0.728	0.521
		MLP	32.256	0.878	0.770	60.670	0.635	0.399
		KRR	51.152	0.653	0.423	61.573	0.627	0.381
Crystal stability	***LE***	**XGBoost**	**1.033**	**0.999**	**0.998**	**3.494**	**0.976**	**0.948**
		AdaBoost	3.187	0.990	0.977	6.724	0.898	0.806
		RF	4.281	0.980	0.958	5.497	0.933	0.870
		MLP	3.124	0.989	0.978	6.416	0.917	0.823
		KRR	4.558	0.976	0.952	4.367	0.959	0.918

The best performing results are marked in bold.

For each of the studied 153 HEDMs, the compound name; its molecular formula, diagram, and conformation; as well as the Cambridge Crystallographic Data Center (CCDC) index number and Chemical Abstracts Service (CAS) number are provided in the Table S3 presented in the supplemental information. Details of our calculation methods of various physicochemical parameters and properties are described in our previous reports (Zhang et al., 2016a, 2016b, 2019a, 2019b, 2019c, 2019d; Jiang et al., 2018; He et al., 2017).

In each ML process for the prediction of *D*, *p*_*C-J*_, *Q*_*max*_, *T*_*d*_, and *LE*, the dataset was divided into a training dataset (80%) and a test dataset (20%), in which stratified sampling was conducted to reduce sampling error and to improve the performance of ML.

#### Feature descriptors

All calculated physicochemical parameters were classified into crystal level and molecular level groups. The *crystal level parameters* include space group, number of molecules in one primitive cell, packing types, packing coefficient (PC), ρ, ρ_N_, OB, intermolecular HB count, intermolecular HB strength, intermolecular HB length, and in-crystal mixture with hydrogen-rich molecules or energetic molecules. The *molecular level parameters* include shape of molecular backbones; molecular weight (MW); intramolecular bond length; intramolecular bond strength; number of critical functional groups, such as –NO_2_, –NH_2_, –OH, –CH_3_, and –N_3_; as well as the distribution of detonation products, including gaseous CO_2_, H_2_O, N_2_, O_2_, and NH_3_ and solid C. Although part of the calculated data for 118 HEDMs was mentioned in our recent studies (Li et al., 2020a, 2020b, 2020c), in the present work, the calculated data of physicochemical parameters for these compounds were significantly increased and further DFT calculations for additional 35 HEDMs were performed to enlarge the dataset.

Among the 5,941 intermolecular HB and intramolecular bonds studied, the strongest intermolecular HB for each HEDM and its weakest intramolecular chemical bond were screened out and used as feature descriptors of the HEDM compound (Tables S1 and S2; supplemental information). Eventually, 28 types of feature descriptors were selected for the following ML study.

**Figure 3:**
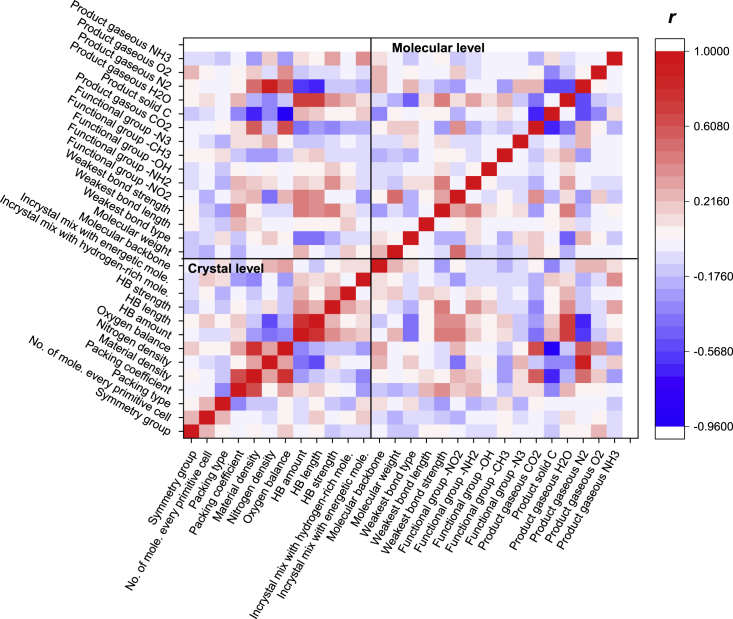
Heatmap of Pearson correlation coefficient of selected features

Furthermore, Pearson correlation coefficient matrices were calculated to identify the positive and negative correlations between pairs of selected features, as shown in Figure 3. The low linear correlations for the feature descriptors indicate that redundant and irrelevant features were not included in current study, which helps improve ML performance.

#### Model training

An appropriate model and its optimized hyperparameters constitute crucial preconditions for the successful application of ML in molecular and materials research. Herein, five representative ML models, including extreme gradient boosting regression tree (XGBoost), adaptive boosting regressor (AdaBoost), RF, multi-layer perceptron (MLP), and KRR, were employed.

Subsequently, grid search method was utilized to optimize the hyperparameters of the five models and 5-fold cross-validation method was used to evaluate the error metrics of each model. Herein, the 5 folds were obtained by random division of the training dataset. The cross-validation procedure was carried out 5 times, and the one with the best error metrics aided in the determination of the final hyperparameters of this model. In the entire training process, the grid search and cross-validation loop were conducted for more than 12,500 times in total and 25 sets of optimal hyperparameters in predicting *D*, *p*_*C-J*_, *Q*_*max*_, *T*_*d*_, and *LE* were eventually determined for the 5 models, as shown in Figure 1. While training the MLP model, the fingerprints were standardized, and the Rectified Linear Unit (ReLU) activation function was used. The hidden layer size is (3, 3) in predicting *D*, *Q*_*max*_, and *LE*, and is (3, 2) in predicting of *T*_*d*_ and *p*_*C-J*_. While training the KRR models, the polynomial kernel function was used in the prediction of *D*, *p*_*C-J*_, *Q*_*max*_, and *T*_*d*_, and the linear kernel function was used in the prediction of *LE*. The parameters were optimized using the grid search method.

#### Model inference and validation

**Figure 4:**
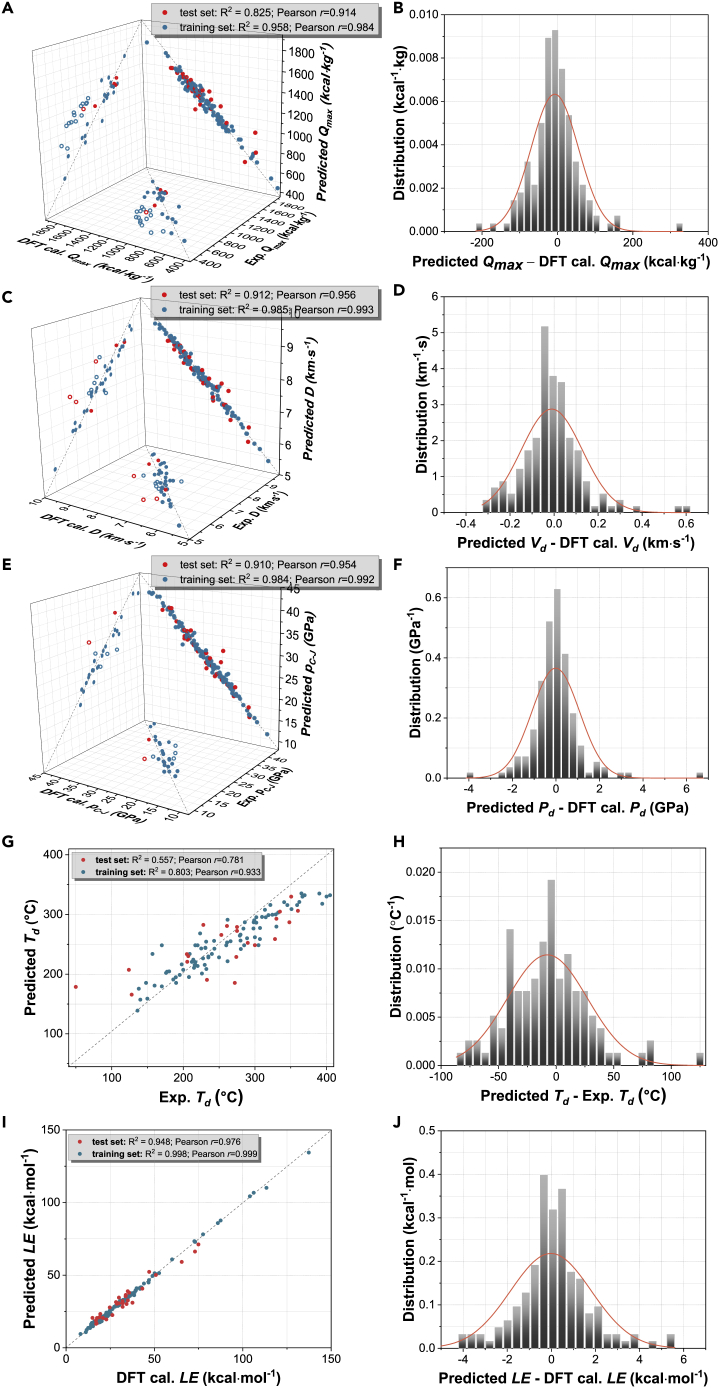
Performance of XGBoost model in predictions of detonation and stability properties of HEDMs as well as experimental validation (A–J) (A) Predicted values of *Q*_*max*_, error metrics, and experimental validation. (B) Distribution of *Q*_*max*_ residuals between XGBoost prediction and DFT calculation. (C) Predicted values of *D*, error metrics, and experimental validation. (D) Distribution of *D* residuals between XGBoost prediction and DFT calculation. (E) Predicted values of *p*_*C-J*_, error metrics, and experimental validation. (F) Distribution of *p*_*C-J*_ residuals between XGBoost prediction and DFT calculation. (G) Predicted values of *T*_*d*_ and their error metrics. (H) Distribution of residuals between XGBoost prediction and experimental measurement. (I) Predicted values of *LE* and their error metrics. (J) Distribution of residuals between XGBoost prediction and DFT calculation. Solid spheres of experimental data are for the densely pressed samples with ρ ≥ 95% ρ_max_, and the open circles are for those compounds with ρ < 95% ρ_max_. Residuals of all XGBoost predictions are shown in Figure S1.

To evaluate the robustness of the above-identified hyperparameters and the performance of the five trained models, herein, the prediction errors were estimated by using three scoring metrics, namely, root-mean-square error (RMSE), Pearson correlation coefficient (*r*), and coefficient of determination (*R*^*2*^) and then the distribution of the residuals of the predictions was compared with the normal distributions. The prediction errors were calculated independently for the training set and test set, as presented in Table 1. The performances of the five models in the prediction of *D*, *p*_*C-J*_, *Q*_*max*_, *T*_*d*_, and *LE* are plotted in Figures S1–S21 (Supplemental information), wherein XGBoost exhibits the best performance. The XGBoost predictions of all five properties of the training set and the test set and the distribution of the prediction residuals of the entire set are shown in Figure 4.

##### Detonation performance characterized based on heat of explosion, detonation velocity, and detonation pressure

The motivation of the current work is to explore the optimal design of advanced HEDMs with simultaneous high detonation performance and high stability, so we consider only the maximum detonation performance, which is calculated at the maximum theoretical density for each of the 153 compounds studied.

For the prediction of *Q*_*max*_, XGBoost model shows the best agreement with the results calculated by the DFT method for the training set (*RMSE* = 49.69 kcal·kg^−1^, *r* = 0.98, and *R*^2^ = 0.96) and the test set (*RMSE* = 99.99 kcal·kg^−1^, *r* = 0.91, and *R*^2^ = 0.82), as summarized in Table 1. Comparative analysis of the distributions of the residuals of the predicted *Q*_*max*_ of the five models, as shown in Figures 4B and S2–S5, indicates that XGBoost prediction residuals present the closest distributions to normal distributions. Furthermore, a simple statistic showed that 74.5% of the XGBoost-predicted *Q*_*max*_ was comparable to the DFT results with a relative error less than 5%, and 93.5% of the XGBoost predictions showed a relative error below 10%, thereby confirming the accuracy of the XGBoost prediction. Compared with a previous RF prediction of *Q*_*max*_ (*R*^2^ = 0.99 for training set and *R*^2^ = 0.93 for test set), current metrics (*R*^2^ = 0.96 for training set and *R*^2^ = 0.82 for test set) are slightly lower; however, our dataset of 153 HEDMs is much larger than the dataset of 41 HEDMs reported by Hong and coworkers (Kang et al., 2020). Our metrics are much better than the KRR predictions of *Q*_*max*_ for 109 energetic compounds, wherein *r* = 0.88 and *R*^2^ = 0.88 for the training set and *r* = 0.79 and *R*^2^ = 0.76 for the test set (Elton et al., 2018).

Regarding the prediction of *D*, all the five studied models exhibited pretty good performance, with all the values of *RMSE* smaller than 0.3 km s^−1^, all values of *r* higher than 0.94, and all values of *R*^*2*^ higher than 0.86, as presented in Table 1. Among the five models studied (Figures S6–S9), XGBoost model reproduced the best agreement with DFT calculations for both the training set and the test set. The scoring metrics for the training set are *RMSE* = 0.10 km·s^−1^, *r* = 0.99, and *R*^2^ = 0.99, and those for the test set are *RMSE* = 0.23 km·s^−1^, *r* = 0.96, and *R*^2^ = 0.91. Figure 4C shows an accurate prediction of *D* by using XGBoost model, wherein 98% of the predicted values have a relative error of <5% with respect to the DFT calculations and 100% of the predicted values have a relative error of <10%. Figure 4D presents a distribution of the residuals of the XGBoost predictions, which satisfactorily follows normal distributions. The currently obtained value of *r* is comparable to a previous ANN prediction of *D* for 65 explosive compounds and compositions, wherein, *r* = 0.978 for training set and 0.985 for test set (Chandrasekaran et al., 2019). The currently obtained values of *R*^2^ and *RMSE* are close to another prediction of 54 nitrogen-rich energetic compounds obtained by least square support vector machine (LS-SVM) method, in which *RMSE* = 0.17 km·s^−1^, *r* = 0.96 for the training set and *RMSE* = 0.17 km·s^−1^, *r* = 0.97 for the test set (Wang et al., 2014). Our metrics are overall better than those of the previous studies as follows: the decision tree boost predictions of *D* for 106 ideal explosives and 231 non-ideal explosives (*RMSE* = 0.41 km·s^−1^, *r* = 0.94 for training set and *RMSE* = 0.34 km·s^−1^, *r* = 0.93 for test set) (Gupta et al., 2016); the partial least squares regression (PLSR) predictions of *D* for 92 ideal, 84 non-ideal, and 68 non-explosive chemicals (*RMSE* = 0.74 km·s^−1^, *r* = 0.87 for training set and *RMSE* = 0.28 km·s^−1^, *r* = 0.96 for test set) (Gupta et al., 2015); the neural network (NN) predictions of *D* for 416 molecules (*RMSE* = 0.27 km·s^−1^, *r* = 0.86, *R*^2^ = 0.93 for test set) (Barnes et al., 2018); and the KRR predictions of *D* for 109 HEDMs (*r* = 0.94 and *R*^2^ = 0.89 for the training set and *r* = 0.91 and *RMSE* = 0.25 km·s^−1^, *r* = 0.91, *R*^2^ = 0.82 for the test set) (Elton et al., 2018).

For the prediction of *p*_*C-J*_, XGBoost model exhibited the best performance among the five models studied herein (Figures S10–S13, Supplemental information), with *RMSE* being the lowest and *r* and *R*^2^ being closest to 1. The scoring metrics are *RMSE* = 0.82 GPa, *r* = 0.99, and *R*^2^ = 0.98 for the training set and *RMSE* = 1.79 GPa, *r* = 0.95, and *R*^2^ = 0.91 for the test set. Figure 4E illustrates the accuracy of the XGBoost prediction of *p*_*C-J*_, wherein 88.2% of the predicted values showed a relative error of <5% referring to the DFT calculation and 97.4% of the predicted values were within a relative error of <10%. Figure 4D demonstrates that the distribution of the residuals of XGBoost prediction approximately follows normal distributions. Our scores are overall more satisfactory than those achieved in the previous studies, such as the PLSR and logistic regression predictions of *p*_*C-J*_ for 92 ideal, 84 non-ideal, and 68 non-explosive compounds (the best scores being *RMSE* = 5.45 GPa, *r* = 0.85 for training set and *RMSE* = 2.53 GPa, *r* = 0.93 for test set) (Gupta et al., 2015); the NN predictions of *p*_*C-J*_ for 416 energetic molecules (*RMSE* = 2.15 GPa, *r* = 0.93, *R*^2^ = 0.87 for test set) (Barnes et al., 2018); and the KRR predictions of *p*_*C-J*_ for 109 HEDMs (*r* = 0.67 for the test set) (Elton et al., 2018).

To validate the predictions of the detonation parameters, we searched 112 experimental detonation parameters from comprehensive literature survey, but we only compared our predicted values with corresponding experimental values measured for the compounds close to its theoretical maximum density: ρ ≥ 95% ρ_max_, wherein ρ_max_ is the maximum theoretical density as determined by X-ray crystallography. Figures 4A–4E and Table S3 (supplemental information) present satisfactory agreement between the XGBoost predictions and 83 experimental data reported for densely pressed samples with ρ ≥ 95% ρ_max_, as marked in solid. Another 29 experimental data of the loosely pressed samples of HEDMs (ρ < 95% ρ_max_) are also presented in Figure 4, as marked in open circles. As expected, our XGBoost predictions (for compounds with ρ = ρ_max_) are generally higher than the corresponding experimental measurements (for samples with ρ < 95% ρ_max_). Thus the XGBoost models in predictions of *Q*_*max*_, *D*, and *p*_*C-J*_ were validated.

##### Molecular stability characterized by decomposition temperature

The decomposition temperature (*T*_*d*_) herein refers to on-set temperature of the exothermic curve, and it is employed to represent the critical temperature at which the molecule starts to lose its stability.

For the prediction of molecular stability characterized by *T*_*d*_, all five models studied showed relatively poor performance compared with the predictions of detonation properties Among the five models, RF exhibited the best performance for the training set (*RMSE* = 21.23°C, *r* = 0.97, and *R*^2^ = 0.90), whereas XGBoost (*RMSE* = 52.07°C, *r* = 0.78, and *R*^2^ = 0.56) was the best performing model for the test set, as presented in Table 1. The distribution of residuals between XGBoost prediction and experimental measurement was close to normal distribution, as shown in Figures 4H and S14–S17. Figure 4G presents that 57.8% of the XGBoost predictions of *T*_*d*_ have a relative error of <10% with respect of the experimental results and 90.8% of the predictions were found to be in the range of a relative error of below 20%. A previous ANN prediction of *T*_*d*_ of energetic co-crystals showed high metrics with R^2^ = 0.98. However, this prediction was based on a limited dataset (19 training data and 6 test data), which is too small to deliver convincing statistics (Fathollahi and Sajady, 2018). Similarly poor performance was also observed in the prediction of experimental power conversion efficiency of organic solar cell devices, where RF was identified to be the best performing model, yet with a low *r* value of 0.70 (Wu et al., 2020).

The quality of the *T*_*d*_ dataset is the fundamental reason for the relatively low performance of the current prediction of *T*_*d*_ and the linearity problem between residual and target data of the trained models (Figure S1D). Among the 153 HEDMs studied in this work, only 109 were found in the literature to have reported *T*_*d*_. These collected *T*_*d*_ values from the literature search were measured using different techniques, using different parameters, and by different investigators. Among the 109 *T*_*d*_ values, 85 were recorded by the on-set temperature of differential scanning calorimetry (DSC) method, 9 were determined by the on-set temperature of differential thermal analysis method, and for the remaining 15 compounds, the *T*_*d*_ values were reported, but the measuring methods were not provided. Besides the application of various techniques, the used heating rate, sample weight, carrier gas flow rate, and other parameters were quite dispersed, all significantly affecting the results and leading to deviations in the reported *T*_*d*_. For example, the different heating rate can shift the location of the exothermic peak in DSC thermograms by up to 49°C and different compositions may change the *T*_*d*_ of TNT by up to 73°C (Li et al., 2019). For each of the 109 *T*_*d*_ used, the corresponding measuring method (if known) is listed in Table S3 (Supplemental information).

The current study indicates that a unified standard for thermal analysis of HEDMs is strongly needed, and this will help experimenters generate more transferable and more reliable *T*_*d*_ values. Then, scientists will have more transferable and more reliable values to evaluate the molecular stability of various HEDMs. Also, ML prediction of *T*_*d*_ of HEDMs is expected to have much-improved performance.

##### Crystal stability characterized by lattice energy

For the prediction of crystal stability characterized by *LE*, XGBoost model exhibited the best performance among the five models studied (Figures S18–S21; Supplemental information). The scoring metrics are *RMSE* = 1.03 kcal·mol^−1^, *r* = 1.00, and *R*^2^ = 1.00 for the training set, and *RMSE* = 3.49 kcal·mol^−1^, *r* = 0.98, and *R*^2^ = 0.95 for the test set. Figure 4I shows a high-level reproduction of the DFT calculation of *LE*, wherein 69.3% of the XGBoost predicted values showed a relative error of <5% and 87.6% of the predicted values were within a relative error of <10%. Figure 4J indicates that the distribution of the XGBoost prediction residuals approximately follows normal distributions.

### Physics insight from machine learning

#### Main features in determining detonation performance, molecular stability, and crystal stability

**Figure 5:**
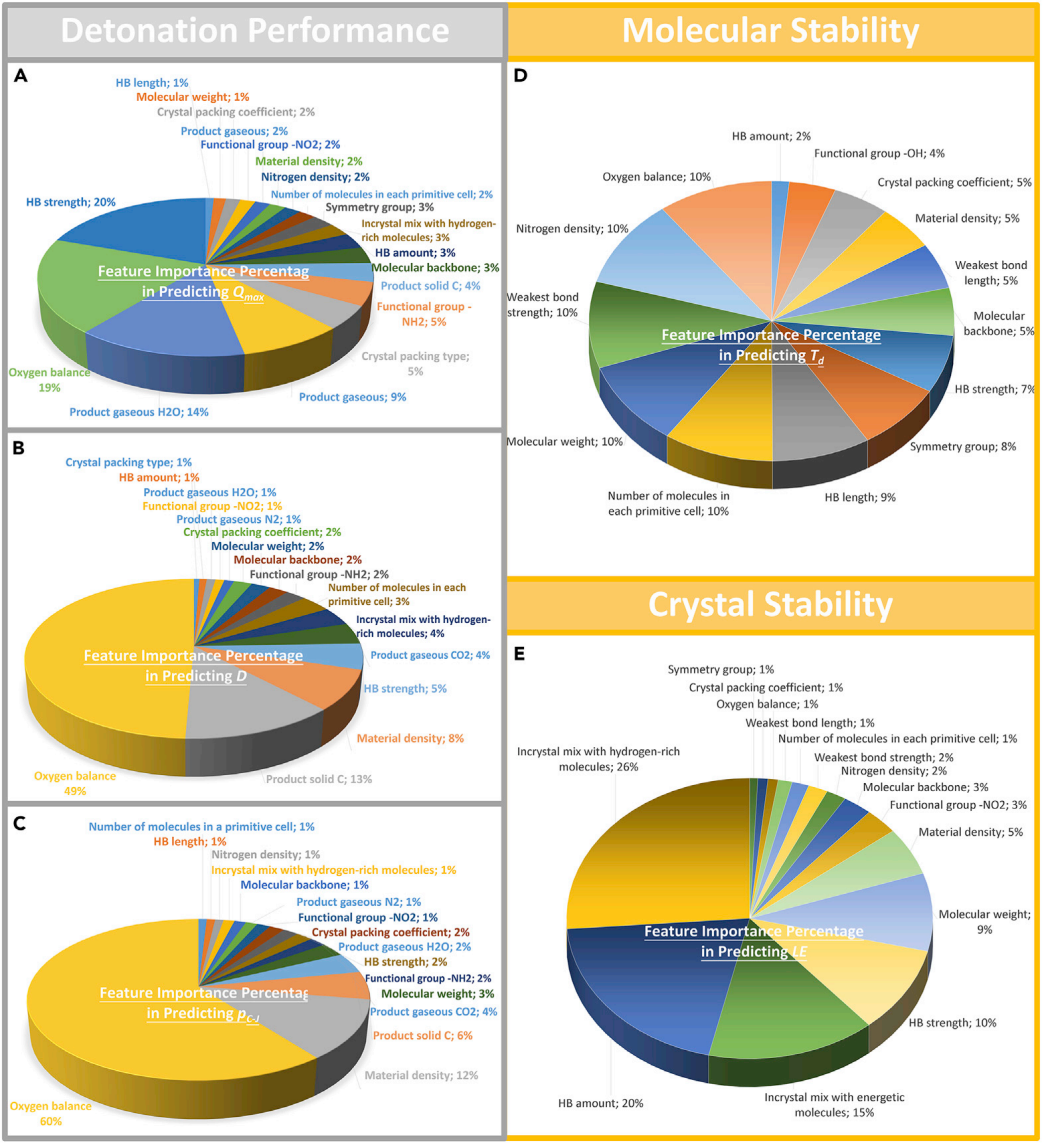
Pie chart of importance ranking of feature descriptors in predictions of detonation and stability properties of HEDMs (A) *Q*_*max*_; (B) *D;* (C) *p*_*C-J*_; (D) *T*_*d*__*;*_ and (E) *LE*.

After the training and evaluation of the five models used in the prediction of *D*, *p*_*C-J*_, *Q*_*max*_, *T*_*d*_, and *LE*, the best performing XGBoost model was eventually selected for each property to conduct feature importance analysis. In this work, the gain-type algorithm in XGBoost regression was utilized to rank feature importance. The features leading to higher average gain values in decision trees are considered more important. The resulting importance was characterized by percentage, and the rankings of the importance of the features in determining the detonation and stability properties are shown in Figure 5. Another feature importance ranking, which is quantified based on the magnitude of Pearson correlation coefficients, is presented in Table S4 (Supplemental information) for comparative analysis, although the latter method is mostly sensitive to linear dependencies.

##### Feature importance ranking in XGBoost predictions of heat of explosion, detonation velocity, and detonation pressure

Detonation performance, which is usually characterized by *Q*_*max*_, *D*, and *p*_*C-J*_, is one the most valued properties in an HEDM evaluation. Figures 5A–5C present the importance ranking of feature descriptors in the determination of detonation properties of HEDMs.

The common characteristic of the top five important features in predictions of *Q*_*max*_, *D*, and *p*_*C-J*_, are OB and the presence of gaseous CO_2_ in the reaction products. Figure 5 illustrates that OB ranks as the most important feature to predict *p*_*C-J*_ and *D* and as the second important feature to determine *Q*_*max*_, acquiring 60%, 49%, and 19% in the feature importance pie chart in determining *p*_*C-J*_, *D*, and *Q*_*max*_, respectively. Currently the obtained data-driven conclusion is well consistent with known professional knowledge, that is, OB describes the extent to which a certain HEDM could be oxidized and significantly affects the amount of energy released during the detonation (Figure S22; supplemental information). Figure 5 shows that the presence of gaseous CO_2_ in the reaction products plays as the fourth importance role in predicting *Q*_*max*_ and *p*_*C-J*_, and as the fifth most important parameter in predicting *D*, achieving 9%, 4%, and 4% of the importance percentage, respectively. Pearson correlation coefficients listed in Table S4 indicate that the presence of gaseous N_2_ in the reaction products is also important to detonation properties. These results are consistent with our recent knowledge-driven findings (Li et al., 2020a, 2020b, 2020c) that the generation of large amount of CO_2_ and N_2_ gas is conducive to the improvement of *Q*_*max*_, *D*, and *p*_*C-J*_ of HEDMs. Furthermore, it was found that molecules, with high MW value and energetic rings connected via bridge bonds or with non-planer heterocyclic or cage-like backbones, are responsible for the better *Q*_*max*_ of these molecules, whereas they have only a minimal influence on *D* and *p*_*C-J*_ (Figure S27; Supplemental information).

In particular, the presence of gaseous H_2_O in the products ranked third in determining *Q*_*max*_; the presence of solid C ranked second in determining *D* and ranked third to predict *p*_*C-J*_, as presented in Figure 5. Pearson correlation coefficients presented in Table S4 (Supplemental information) indicate that the contribution of the presence of gaseous H_2_O in the products is negative for obtaining large value of *Q*_*max*_, and the contribution of the presence of solid C in the products has also negative effect on obtaining large values of *D* and *p*_*C-J*_. These results are consistent with the recent finding (Li et al., 2020a, 2020b, 2020c) that high proportion of H_2_O in the products leads to a lower *Q*_*max*_ and high content of solid carbon clusters in the detonation products, which are obtained due to the oxygen deficiency in the structure of an HEDM, leads to a drastic decrease in *D* and *p*_*C-J*_ of HEDMs.

Noteworthy, crystal features, such as the highest strength of intermolecular HB, the material density ρ, and the HB amount (characterized by the area of HB on the Hirshfeld surface of each individual molecule), are crucial for the determination of the detonation properties. Previous research noticed the importance of ρ parameter in the prediction of the detonation properties, whereas the other two features were often overlooked. HB amount and HB strength determine the interspecies association distribution, interaction strength, packing compactness, and energy level of the compound, and are therefore important to the energy that could be released in detonation. Both high HB amount and high HB strength of an HEDM could lead to a significant reduction in its detonation performance (Figures S23 and S24; Supplemental information), consistent with the negative Pearson correlation coefficients listed in Table S4 (Supplemental information). Based on our current study, the importance of crystal characteristics to detonation properties of HEDMs needs to be re-understood comprehensively.

##### Feature importance ranking in XGBoost prediction of molecular stability

Higher thermal stabilities usually result in HEDMs with lower sensitivities, which are safer to handle. *T*_*d*_ is an important characterization index of thermal stability of compounds at the molecular level, and a search for high-performance thermostable HEDMs, with *T*_*d*_ higher than that of hexanitrostilbene (HNS), i.e., *T*_*d*_ > 330°C, is an active research topic in the field of HEDMs (Rieckmann et al., 2001; Klapötke, 2015).

Figure 5D exhibits the description of the importance ranking of feature descriptors used for the determination of *T*_*d*_. In contrast to the detonation properties, the importance percentage toward determination of *T*_*d*_ is rather uniform and disperse. Each of the 12 features occupies >5% of the importance percentage. These features are OB, ρ_N_, the weakest/longest intramolecular bond strength/length, MW, molecular backbone, the strongest/shortest intermolecular HB strength/length, number of molecules located in each primitive cell, and symmetry group. Among them, the molecular features are more important than the crystal features.

At the molecular level, OB is important in determining *T*_*d*_, i.e., higher OB results in lower *T*_*d*_, which contradicts the requirement of high detonation performance (Table S4 and Figure S22; Supplemental information). Second, the weakest intramolecular bond is the trigger of the decomposition of HEDMs and plays a crucial role to determine *T*_*d*_ (Table S4 and Figure S26; supplemental information). Third, current result indicates that large MW and bridged or non-planar molecular backbone significantly improve *T*_*d*_, in a way similar to the improvement of *Q*_*max*_ (Table S4 and Figure S27; supplemental information).

At the crystal level, HB amount and HB strength have been proved to be important in predicting *T*_*d*_, as shown in Figure 5D and Table S4 (supplemental information). These features determine the crystal packing force, which in turn influences the activation energy of the molecule decomposition. Figures S23 and S24 (Supplemental information) exhibit that the requirement of high HB amount and high HB strength for high *T*_*d*_ contradicts the high detonation performance.

##### Feature importance ranking in XGBoost prediction of crystal stability

Crystal stability of HEDMs refers to the extent to which the crystals can sustain cohesion as solids against sublimation into separate molecules. Herein, *LE* was employed to characterize crystal stability, which is defined as the total energy difference between the constituent ions in the free state and the crystal form. A comparable measurement of *LE* from experiment is the sublimation temperature. However, the reported data for the sublimation of HEDMs were extremely limited, because many of the HEDMs would not sublime but would melt. Therefore, we used melting point as an alternative value to compare with *LE*, validating the roughly positive correlation between *LE* and crystal stability, as shown in Figure S1 (supplemental information).

Figure 5E presents the importance ranking of feature descriptors in determining *LE*. In contrast to *T*_*d*_, the crystal features to determine *LE* are more important than the molecular features, which is consistent with the ranking from Pearson correlation coefficients listed in Table S4 (Supplemental information). Among them, HB=relevant features are the most important to determine *LE*. The in-crystal mixing with hydrogen-rich molecules, HB amount, and HB strength together contribute 56% of the feature importance percentage. Having a higher HB amount and higher HB strength is advantageous to improve *LE*, enabling corresponding HEDMs to achieve higher thermal stability. However, they contradict with the requirement of high detonation performance (Figures S23 and S24; supplemental information).

Third ranked feature is the in-crystal mixing with of energetic molecules, which takes 15% of the importance percentage. The importance of this feature to determine *LE* confirms the rationality of co-crystallization strategy in the promotion of thermostability of HEDMs.

From the perspective of molecular level features, MW and molecular backbone are important in determining *LE*. HEDMs composed of bridged molecules with high MW are very likely to show higher *LE*. For example, the compounds such as 3,5-dinitro-*N*,*N′*-bis(2,4,6-trinitrophenyl)pyridine-2,6-diamine, 5,5′-ethane-1,2-diylbis(1H-tetrazole), and 3,3'-(1,4-phenylenediethene-2,1-diyl)bis-(2,4,6-trinitroaniline)-*N*,*N*-dimethylformamide solvate, with their energetic rings connected with NH/NH_2_/CH/CH_2_ bridge bonds, all have relatively high *LE*. 3,5-Dinitro*-N*,*N′*-bis(2,4,6-trinitro-phenyl)pyridine-2,6-diamine (MW = 621.35), constituted by three energetic rings and two “–NH–” bridges, has a high *LE* = 51.01 kcal·mol^−1^ that is 2.4 times higher than that of TNT (single energetic ring, MW = 227.20). Similarly, the compounds with non-planar heterocyclic or cage-like backbones, such as HMX (MW = 296.2) and CL-20 (MW = 438.2), have high *LE* values that are 1.8 and 1.4 times larger than that of TNT.

#### Guidelines for balancing performance and stability

From feature importance ranking and data statistics, herein, the features were classified into the two distinctive types: (1) contradictory features and (2) non-contradictory features, to balance the detonation performance and stability of HEDMs. Optimal ranges of all found critical features of HEDMs for balancing detonation performance and stability are presented in Table 2.

**Table 2:** Optimal ranges of critical features of HEDMs for balancing detonation performance and stability

Features	Optimal ranges
Oxygen balance (OB)	−60 < OB < 0%
HB strength σ	HB σ > 2.5 kcal mol^−1^
	2.5 < HB σ < 5.0 kcal mol^−1^ (regardless *Q*_*max*_)
HB amount	Ø
	>90 Å^2^ (regardless *Q*_*max*_)
	>30 Å^2^ (regardless *Q*_*max*_ and *LE*)
Material density (*ρ*)	ρ > 1.73 g cm^−3^
Weakest intramolecular strength (σ)	σ > 70.0 kcal mol^−1^
Molecular weight	>250 Da
Molecular backbone	Bridged backbones
	Non-planar backbones of heterocycles or cages

In our opinion, the OB, HB amount, and HB strength are the main parameters that lead to the performance-stability contradiction of HEDMs (Figures S22–S24; supplemental information). OB = 0% ranks first among all other features in providing the best *p*_*C-J*_ and *D* values, whereas it also ranks first in reducing *T*_*d*_. Considering the detonation properties (*p*_*C-J*_ = 22.35 GPa and *D* = 7.15 km·s^−1^) and detonation temperature (*T*_*d*_ = 225°C) of TNT, as a reference, the optimal range of OB that balances detonation and stability is in the range of −60% < OB < 0%. HB amount seems to be the most significant feature that causes detonation performance-crystal stability contradiction. Abundant HB is the most important requirement of high *LE*; however, high amount of HBs is accompanied with a high proportion of H_2_O in the detonation products, thereby decreasing *Q*_*max*_. Figure S23 (supplemental information) illustrates that there is no intersection of HB amount to simultaneously satisfy high detonation performance and high crystal stability. However, if application of the designed HEDM mostly demands high *p*_*C-J*_ and *D*, regardless of *Q*_*max*_, the optimal range of HB amount can be >90 Å^2^. In case that a high *T*_*d*_ of the designed HEDM is the main objective, whereas high crystal stability is less important, the optimal range for such HEDMs could be further extended to be HB amount >30 Å^2^. High HB strength leads to a reduced total energy of the system, thereby decreasing *Q*_*max*_ of HEDMs. However, high HB strength is important for HEDMs to reach high *LE* and high *T*_*d*_. To balance detonation performance and crystal stability, the recommended range of the strength of the strongest HB is in the range of 2.5 < HB σ < 5.0 kcal·mol^−1^. However, HB strength has a limited relevance to *p*_*C-J*_ and *D*. If application of the designed HEDM mostly demands high *D* and *p*_*C-J*_, regardless of *Q*_*max*_, the optimal range of HB strength is recommended to be HB amount >2.5 kcal·mol^−1^.

In this work, it was found that ρ and the weakest intramolecular bonds are non-contradictory features in balancing detonation performance and crystal stability. Although a high ρ is always a pursuit for achieving high detonation performance of HEDMs, it has little relevance to *T*_*d*_ or *LE* (Figure S25; supplemental information). Considering the detonation performance of TNT as a reference, the recommended range is ρ > 1.73 g·cm^−3^. Strong intramolecular bonds and high MW are important for HEDMs to improve both molecular stability and crystal stability. Theoretically, stronger bond decreases the energy level of energetic molecules, leading to a small energy difference between the reactants and the products, and thereby reduction in the detonation performance of HEDMs. Yet, feature importance analysis shows that the strength of intra-molecular bonds does not significantly influence detonation properties. Therefore, strengthening the intramolecular bonds can be a potential way to balance the detonation and stability of HEDMs, and the recommended range is σ > 70.0 kcal mol^−1^ (Figure S26; supplemental information).

Very importantly, it was found that the design of molecules with high MW, bridged backbones, or non-planar heterocyclic or cage-like backbones could lead to simultaneous improvement in the detonation performance and the stability of HEDMs. Furthermore, co-crystallization is an effective strategy for improving the thermal stability of HEDMs.

## Discussion

In this study, ML methodology was employed to better understand intricate causality of the detonation performance-stability contradiction, and to facilitate more efficient rational design of advanced HEDMs, by providing data-driven recommendations of critical parameters. More specifically, quantum mechanics method was used to calculate 21,648 physicochemical parameters, detonation properties, and stability properties of 153 reported HEDMs, which were then used as the input for training of five ML models, i.e., XGBoost, AdaBoost, RF, MLP, and KRR. Stratified sampling was employed to classify training set and test set by a ratio of 4:1, and grid search and cross-validation loop were conducted for more than 12,500 times in total to optimize the hyper-parameters in predicting *D*, *p*_*C-J*_, *Q*_*max*_, *T*_*d*_, and *LE* of the HEDMs. By evaluating the scoring metrics, the distribution of prediction residuals, and the deviation from experimental data, XGBoost model was proved to exhibit the best performance in predictions of all five properties of HEDMs.

Furthermore, feature importance analysis was carried out by two methods, i.e., the gain-type algorithm in XGBoost regression and the magnitude of Pearson correlation coefficients. The contradictory features and non-contradictory features in balancing detonation performance and stability of HEDMs were subsequently screened out. The optimal range of key features, which facilitated the optimal balance between the two contradictory factors, was identified to be (1) −60 < OB < 0%, (2) HB strength >2.5 kcal·mol^−1^, (3) ρ > 1.73 g·cm^−3^, (4) the weakest intramolecular strength >70.0 kcal·mol^−1^, (5) and MW > 250 Da. Design of molecules with large MW, bridged backbones, and non-planar backbones of heterocycles or cages, are generally favorable in improving both detonation performance and stability of HEDMs.

### Limitations of the study

There is a linearity problem between residual data and target data in the prediction of decomposition temperature *T*_*d*_. We tried several methods to fix this problem, for example, adjusting model parameters, using standardized fingerprints, and using Box-Cox transformation for target data. However, all these attempts failed. We later tried to artificially modify the trained models according to the fitted linear relation between the predicted data and the target data of the training set. Although the linearity problem in XGBoost, AdaBoost, and RF models was finally fixed, artificial fixing of the trained model may confuse the readers and cause misleading. We finally decided to respect the truth and use the originally trained model, instead of the artificially fixed ones.

As we have discussed in the section molecular stability characterized by decomposition temperature, the *T*_*d*_ dataset was searched from massive literature. The quality of the *T*_*d*_ dataset is the fundamental reason that various models cannot learn *T*_*d*_, leading to the linearity problem and the low scoring metrics in predicting *T*_*d*_. The current study indicates that a unified standard for thermal analysis of HEDMs is strongly needed, and this will help experimenters generate more transferable and more reliable *T*_*d*_ values. Then, scientists will have more reliable values to evaluate the stability of various HEDMs. Also, ML on the stability of HEDMs is expected to have much-improved performance.

### Resource availability

#### Lead contact

Further information and requests for resources should be directed to the lead contact, Lei Zhang (zhang_lei@iapcm.ac.cn).

#### Materials availability

This study did not generate new unique reagents.

#### Data and code availability

The source data that support the findings of this study and the used codes are available from the corresponding authors upon reasonable request.

## Methods

All methods can be found in the accompanying transparent methods supplemental file.

## Supplemental information

Document S1. Transparent methods, Figures S1–S27, and Tables S3 and S4

Table S1. List of optimized length (Å) and calculated strength (kcal·mol^−1^) of each intramolecular bond and intermolecular hydrogen bond (HB) in all 153 HEDMs studied, related to Figure 1 . The first weakest intramolecular bonds and the first strongest intermolecular hydrogen bonds in each compound are marked by red colors, and the second weakest intramolecular bonds are marked by blue.

Table S2. All input parameters for machine learning, including calculated physicochemical parameters and experimental decomposition temperatures, related to Figure 1

## Footnotes

Supplemental Information can be found online at https://doi.org/10.1016/j.isci.2021.102240.
