# Supplementary material for: Applying machine learning to balance performance and stability of high energy density materials
Source: iScience. 2021 Feb 26;24(3):102240. doi: 10.1016/j.isci.2021.102240 (PMC7957118; doi:10.1016/j.isci.2021.102240)
Supplement: Document S1. Transparent methods, Figures S1–S27, and Tables S3 and S4 [file mmc1.pdf]

iScience, Volume *24*

Supplemental information

Applying machine learning to balance performance and stability of high energy density materials

Xiaona Huang, Chongyang Li, Kaiyuan Tan, Yushi Wen, Feng Guo, Ming Li, Yongli Huang, Chang Q. Sun, Michael Gozin, and Lei Zhang

Supplementary Information

Table of contents

Transparent methods..........2
Table S3. Collected experimental data of heat of explosion Q_{max}, detonation velocity D, and detonation pressure p_{C-J}, decomposition temperature T_d, and melting temperature of the 153 HEDMs studied in this work 3
Table S4. Feature importance ranking by the magnitude of Pearson correlation coefficients 19
Figure S1. XGBoost prediction residuals for Q_{max}, D, p_{C-J}, T_d, and LE..........21
Figure S2. Prediction of heat of explosion Q_{max} with AdaBoost model 22
Figure S3. Prediction of heat of explosion Q_{max} with RF model 22
Figure S4. Prediction of heat of explosion Q_{max} with MLP model..........23
Figure S5. Prediction of heat of explosion Q_{max} with KRR model..........23
Figure S6. Prediction of detonation velocity D with AdaBoost model 24
Figure S7. Prediction of detonation velocity D with RF model 24
Figure S8. Prediction of detonation velocity D with MLP model..........25
Figure S9. Prediction of detonation velocity D with KRR model..........25
Figure S10. Prediction of detonation pressure p_{C-J} with AdaBoost model..........26
Figure S11. Prediction of detonation pressure p_{C-J} with RF model..........26
Figure S12. Prediction of detonation pressure p_{C-J} with MLP model..........27
Figure S13. Prediction of detonation pressure p_{C-J} with KRR model..........27
Figure S14. Prediction of decomposition temperature T_d with AdaBoost model..........28
Figure S15. Prediction of decomposition temperature T_d with RF model..........28
Figure S16. Prediction of decomposition temperature T_d with MLP model 29
Figure S17. Prediction of decomposition temperature T_d with KRR model 29
Figure S18. Prediction of lattice energy LE with AdaBoost model..........30
Figure S19. Prediction of lattice energy LE with RF model..........30
Figure S20. Prediction of lattice energy LE with MLP model 31
Figure S21. Prediction of lattice energy LE with KRR model 31
Figure S22. Optimal range of oxygen balance in balancing detonation performance and stability of HEMDs...32
Figure S23. Optimal range of HB amount in balancing detonation performance and stability of HEMDs..........33
Figure S24. Optimal range of HB strength in balancing detonation performance and stability of HEMDs 34
Figure S25. Optimal range of density in balancing detonation performance and stability of HEMDs..........35
Figure S26. Optimal range of strength of the weakest intramolecular bond in balancing detonation performance and stability of HEMDs..........36
Figure S27. Optimal range of molecular weight in balancing detonation performance and stability of HEMDs. 37
References 38

Transparent methods

All the 21648 physicochemical parameters, detonation performance parameters and stability properties of 153 HEDMs were calculated directly on a crystal level based on the recently developed supercomputing density functional theory (DFT) software, namely, High Accuracy atomistic Simulation package for Energetic Materials (HASEM)(Zhang et al., 2016), which is adapted to modern supercomputers on the basis of the J parallel Adaptive Structured Mesh applications INfrastructure (JASMIN)(Mo et al., 2010).

The training and evaluation of XGBoost model were performed using the XGBoost package, and those of AdaBoost, RF, MLP, and KRR were performed using the scikit-learn package. Stratified sampling was employed to classify training set and test set by a ratio of 4:1. Grid search and cross-validation loop were conducted to optimize the hyperparameters in predicting detonation velocity *D*, detonation pressure p_{C-J}, heat of explosion $Q_{max,}$ decomposition temperature T_d and lattice energy *LE* of the HEDMs. By evaluating the soring metrics, the distribution of prediction residuals and the deviation from experimental data, the best performing model was selected for feature importance analysis.

Table S3. Collected experimental data of detonation performance (heat of explosion Q_{max}, in kcal/kg, detonation velocity D, in km/s, and detonation pressure $p_{C\text{-}J}$, in GPa), molecule stability (decomposition temperature T_d, in °C), and crystal stability (melting temperature, in °C) of the 153 HEDMs studied in this work. Solid scatters ● are for the densely pressed samples with ρ ≥ 95% $ρ_{max}$, and open circles ○ are for those compounds with ρ < 95% $ρ_{max}$, wherein $ρ_{max}$ is the maximum theoretical density as determined by X-ray crystallography. The value in the bracket of the T_d column is the heating rate, in °C per minute, and the thermal analysis method (if it is recorded in the original experiment) is also presented. **(**Related to Figure 4)

	CSD No.	CAS No.	Detonation performance			Molecule stability		Crystal stability
			Q_{max}	D	$p_{C\text{-}J}$	T_d	method	Melting temperature
1	SEDTUQ	145250-81-3	--	--	--	230.85(Crawford et al., 2007)	DSC	238(decomp)(Cai et al., 2004)
2	NOETNA02	19836-28-3	1248(●)(Meyer et al., 2007)	8.85(●)(Tsyshevsky et al., 2017)	35.5(●)(Dong et al., 1989)	177.4(Liu et al., 2016)	DSC	93.5(Liu et al., 2016)
3	TATNBZ	3058-38-6	935(●)(Akst, 1989)	7.76(●)(Keshavarz, 2008)	26.8(●)(Wang et al., 2006)	366.4(Nair et al., 2007)	DSC	365(Atkins et al., 1986)
4	NTROMA01	75-52-5	1152(●)(Keshavarz, 2012)	6.35(○)(Meyer et al., 2007)	--	390(Taylor et al., 2002)	--	-28.6(Bagryanskaya et al., 1983)
5	SEDTUQ09	145250-81-3	978(●)(Meyer et al., 2007)	8.34(●)(Bemm et al., 1998)	34.0(●)(Bemm and Östmark, 1998)	--	--	--

6	SEDTUQ06	145250-81-3	--	--	--	--	--	--
7	NXENAM01	4185-47-1	1304(●)(Keshavarz, 2005)	8.00(●)(Rothstein et al., 1979)	31.0(●)(Wang et al., 2006)	189.6(10)(Zhang et al., 2018)	DSC	51.32(10)(Zhang et al., 2018)
8	NOEURA	918-99-0	1465(●)(Meyer et al., 2007)	9.00(●)(Rothstein and Petersen, 1979)	--	--	--	185(Kwasny et al., 1980)
9	NABMUY01	28464-24-6	--	8.10(●)(Tsyshevsky et al., 2017)	--	--	--	--
10	PERYTN12	78-11-5	1504(●)(Dong and Zhou, 1989)	8.60(●)(Dong and Zhou, 1989)	35.0(●)(Kamlet et al., 1968)	208(Lee et al., 2002)	DSC	142.2(Lange et al., 2009)
11	ZZZQSC02	606-20-2	795(○)(Keshavarz, 2012)	--	--	285(Lewis et al., 1996)	--	66(Bachman et al., 1958)
12	TNOXYL	632-92-8	844(○)(Keshavarz, 2005)	6.70(●)(Wang et al., 2006)	21.2(●)(Wang et al., 2006)	209(Guo et al., 2006)	DSC	182(Meyer et al., 2016)
13	DNNAPH	605-71-0	724(○)(Keshavarz, 2012)	5.52(●)(Wang et al., 2006)	--	--	--	217(Trotter, 1960)

14	GEMZAZ	55510-04-8	717(○)(Meyer et al., 2007)	7.58(○)(Dong and Zhou, 1989)	30.1(●)(Wang et al., 2006)	215(Khire et al., 2005)	DTA	249(decomp)(Boileau et al., 1985)
15	HNIABZ20	19159-68-3	1420(●)(Headqurters, 1984)	7.31(○)(Keshavarz, 2008)	--	348.1(10)(Zhang et al., 2013)	DSC	215(Leemann et al., 1908)
16	PUTCEM	25243-36-1	980(○)(Meyer et al., 2007)	7.25(●)(Keshavarz, 2008)	--	394.0(5)(Altmann et al., 1998)	--	378(Meyer et al., 2016)
17	NTRGUA03	556-88-7	653(●)(Meyer et al., 2007)	7.98(○)(Keshavarz et al., 2005)	24.5(●)(Hobbs et al., 1993)	230(Antonangeli et al., 2010)	--	232(decomp)(Davis et al., 1925)
18	DNEDAM	505-71-5	1023(●)(Meyer et al., 2007)	8.23(●)(Rothstein and Petersen, 1979)	27.3(○)(Gill et al., 2006)	180.3(Hussein et al., 2018)	DSC	178(Hall et al., 1951)
19	CORYIR	55-63-0	1485(●)(Meyer et al., 2007)	7.59(○)(Meyer et al., 2007)	25.3(○)(Hobbs and Baer, 1993)	50(Kim et al., 2018)	--	14(Altenburg et al., 2009)
20	CIWMEA10	97645-24-4	1516(●)(Keshavarz, 2012)	--	--	226(Sikder et al., 2004)	DSC	100(Singh et al., 2005)

21	QOYJOD	932-64-9	722(●)(Volk et al., 1997)	7.86(●)(Meyer et al., 2007)	31.5(○)(Akst, 1989)	279(Wu et al., 2015)	DSC	270(Schmidt et al., 1965)
22	WEKGUP	25242-76-6	1271(●)(Meyer et al., 2007)	7.77(○)(Meyer et al., 2007)	--	170.0(6)(Licht et al., 1988)	DTA	--
23	DNITBZ02	100-25-4	--	6.50(●)(Wang et al., 2006)	--	--	--	174(Boyer et al., 1959)
24	DNBENZ11	99-65-0	--	6.38(●)(Wang et al., 2006)	--	216.8(Wang et al., 2014)	DSC	90.3(McNeil et al., 2013)
25	ZZZGVU02	121-14-2	763(○)(Keshavarz, 2012)	--	--	280(Colonna et al., 2010)	--	70(Bachman and Vogt, 1958)
26	TNITAN	3698-54-2	1023(○)(Meyer et al., 2007)	--	--	216(decomp)(Dobratz et al., 1985)	--	--
27	CTMTNA03	121-82-4	1340(●)(Akst, 1989)	8.75(●)(Dong and Zhou, 1989)	34.7(●)(Politzer et al., 2011)	--	--	--
28	CTMTNA04	121-82-4	--	--	--	235(10)(Jiao et al., 2014)	DSC	206(10)(Jiao et al., 2014)

29	OCHTET	2691-41-0	--	--	--	--	--	--
30	OCHTET01	2691-41-0	1321(●)(Dong and Zhou, 1989)	9.01(●)(Politzer and Murray, 2011)	37.3(●)(Wang et al., 2006)	--	--	--
31	OCHTET03	2691-41-0	--	--	--	280.3(Gao et al., 2014)	DSC	279(Gao et al., 2014)
32	PUBMUU01	135285-90-4	--	--	--	--	--	--
33	PUBMUU07	135285-90-4	--	--	--	227.6(Gao et al., 2014)	DSC	252(decomp)(Gore et al., 2007)
34	PUBMUU12	135285-90-4	1454(●)(Meyer et al., 2007)	9.38(●)(Politzer and Murray, 2011)	44.1(●)(Keshavarz, 2008)	--	--	--
35	TNBENZ12	99-35-4	947(○)(Keshavarz, 2005)	7.30(●)(Meyer et al., 2007)	21.9(●)(Kamlet and Dickinson, 1968)	305.1(Zeman, 1980)	DTA	106(Kofler et al., 1948)
36	ZZZMUC08	118-96-7	1290(●)(Headqurters, 1984)	6.93(●)(Keshavarz, 2008)	22.5(●)(Kamlet and Dickinson, 1968)	225(10)(Hong et al., 2015)	DSC	80.8(Šarlauskas, 2010)

37	TNIOAN	489-98-5	858(○)(Keshavarz, 2007)	7.30(●)(Meyer et al., 2007)	24.7(●)(Wang et al., 2006)	324.38(20)(Zeman, 1993)	DSC	188(Spencer et al., 1946)
38	QAGBAB	96-91-3	639(○)(Keshavarz, 2007)	--	--	217(5)(Wurzenberger et al., 2020)	DTA	169.9(Meyer et al., 2016)
39	PICRAC12	88-89-1	1032(○)(Rice et al., 2002)	7.57(●)(Wang et al., 2006; Keshavarz, 2008)	27.7(●)(Wang et al., 2006)	274(10)(Hong et al., 2015)	DSC	122(Srinivasan et al., 2006)
40	SAWBUN	129-66-835860-50-5	947(○)(Keshavarz, 2005)	--	--	231(5)(Zeman, 2003)	DTA	228.7(decomp)(Fonger et al., 2014)
41	DATNBZ	1630-08-6	980(●)(Rice and Hare, 2002)	7.52(●)(Politzer and Murray, 2011)	25.9(●)(Kamlet and Dickinson, 1968)	358.96(20)(Zeman, 1993)	DSC	288(Siele et al., 1962)
42	WEKGOJ	78013-51-1	1056(○)(Keshavarz, 2007)	7.47(○)(Meyer et al., 2007)	--	300(Licht and Ritter, 1988)	DTA	162(Licht and Ritter, 1988)
43	GETFIU	4682-03-5	--	6.60(○)(Meyer et al., 2007)	--	157(5)(Fischer et al., 2016)	DSC	--

44	TNPHNT	4732-14-3	840(○)(Keshavarz, 2008)	6.50(●)(Meyer et al., 2007)	--	--	--	151(Leonard et al., 1956)
45	MTNANL01	479-45-8	1450(●)(Headqurters, 1984)	7.57(●)(Meyer et al., 2007)	26.3(●)(Kamlet and Dickinson, 1968)	170(Lee et al., 1986)	DSC	131.5(Kim et al., 2018)
46	HNIDPA	131-73-7	974(○)(Keshavarz, 2005)	7.20(○)(Meyer et al., 2007)	28.8(○)(Wang et al., 2006)	275(10)(Huang et al., 2011)	DSC	254(Huang et al., 2011)
47	GIMBOT	20062-22-0	1360(●)(Headqurters, 1984)	7.06(●)(Politzer and Murray, 2011)	26.2(●)(Headqurters, 1984)	330(2.5)(Rieckmann et al., 2001)	DSC	318(Klapötke et al., 2016)
48	BAKLII	56140-58-0	--	--	--	275(Zhang et al., 2010)	DSC	270(Blanksma, 1908)
49	DACYEL	97217-74-8	--	--	--	--	--	240(Chaykovsky et al., 1990)
50	AFEPUX	134282-42-1	--	--	--	232.0(5)(Klapötke et al., 2016)	DSC	--
51	TIBMUM	39771-28-3	--	--	--	321.6(10)(Li et al., 2003)	DSC	--
52	IKIMIY	436848-40-7	--	--	--	160.0(Averkiev et al., 2002)	--	--

53	TIBMIA	132683-64-8	--	7.9(●)(Du et al., 2013)	28.1(●)(Du et al., 2013)	350.7(10)(He et al., 2013)	DSC	--
54	YEKQAG	194486-77-6	--	7.99(●)(ZHAO et al., 2013)	29.6(●)(ZHAO and LIU, 2013)	345.3(5)(Wang et al., 2014)	DSC	--
55	CIWMAW	52173-59-8	--	--	--	302(Huang et al., 2019)	DSC	300(decomp)(Guillou et al., 2009)
56	KUBVAH	1246853-06-4	--	--	--	--	--	211(Zaitsev et al., 2009)
57	MOCJUK01	4433-16-3	--	--	--	286(Yan et al., 2019)	DSC	242.0(Roháč et al., 2008)
58	HIQBIV	131394-27-9	--	--	--	365.0(5)(Kumar et al., 2018)	DSC	305.0(Kumar et al., 2018)
59	PITGAD	2411964-98-0	--	--	--	314.0(5)(Domasevitch et al., 2019)	DTA	306.0(Domasevitch et al., 2019)
60	DORYOA	1573131-04-0	--	--	--	284(Li et al., 2014)	DSC	250(Li et al., 2014)
61	PITGEH	175788-77-9	--	--	--	298.0(5)(Domasevitch et al., 2019)	DTA	292.0(Domasevitch et al., 2019)
62	HIQBOB	1006545-77-2	--	--	--	205.5(5)(Kumar et al., 2018)	DSC	--
63	SEFVIL	2215034-55-0	--	--	--	253.2(5)(Tang et al., 2017)	DSC	209.0(Tang et al., 2017)

64	BADRAC	1605347-16-7	--	--	--	--	--	241.0(Yin et al., 2015)
65	WACGOW	32255-27-9	--	7.29(●)(Türker, 2012)	--	--	--	231.0(Terrier et al., 1990)
66	GATFEP	2072820-21-2	--	--	--	310.0(5)(Fischer et al., 2016)	DSC	--
67	GATFUF	2072820-20-1	--	--	--	205.0(5)(Fischer et al., 2016)	DSC	--
68	KIQYUH	NA	--	--	--	319.0(5)(Bölter et al., 2018)	DSC	156.0(Bölter et al., 2018)
69	KIQNUW	NA	--	--	--	330.0(5)(Bölter et al., 2018)	DSC	191.0(Bölter et al., 2018)
70	YAHKID	5180-53-0	--	--	--	332.6(Huang et al., 2011)	DSC	220.0(Huang et al., 2011)
71	JOTNOX	1644578-17-5	--	--	--	249.9(5)(Yin et al., 2014)	DSC	203.6(Yin et al., 2014)
72	ONAVEF01	26670-16-6	--	--	--	266.0(5)(Yang et al., 2016)	DSC	250.0(Yang et al., 2016)
73	MUKREQ	1198599-36-8	--	--	--	223.5(10)(Zeng et al., 2009)	DSC	--
74	LUFXUH	1819967-31-1	--	--	--	405.0(10)(Liu et al., 2015)	DSC	--
75	IBOPEW	33491-88-2	--	7.33(●)(Keshavarz, 2007)	--	362.0(decomp)(Zeman et al., 2010)	--	362.0(decomp)(Zeman et al., 2010)

76	ZUQWIT	133502-79-1	--	--	--	299.0(5)(Wei et al., 2015)	DSC	--
77	OSEWEQ	38082-89-2	--	--	--	360.0(5)(Klapötke et al., 2016)	DSC	360(Fried, 1998)
78	OTIBAW	55148-03-3	--	--	--	369.5(10)(Zhang et al., 2017)	DSC	--
79	LEGYII	2134229-83-5	--	--	--	261.2(5)(Yin et al., 2017)	DSC	--
80	GEYRAG	293324-58-0	--	--	--	307.0(5)(Tang et al., 2018)	DSC	304.0(Tang et al., 2018)
81	GEYQUZ	NA	--	--	--	280.0(5)(Tang et al., 2018)	DSC	278.0(Tang et al., 2018)
82	LEGYAA	2134229-85-7	--	--	--	307.2(5)(Yin et al., 2017)	DSC	--
83	GEYREK	NA	--	--	--	328.0(5)(Tang et al., 2018)	DSC	325.0(Tang et al., 2018)
84	KUBVEL	NA	--	--	--	351.0(5)(Li et al., 2019)	DSC	--
85	KUBVOV	NA	--	--	--	261.9(5)(Li et al., 2019)	DSC	--
86	HEVRUV	517-25-9	--	--	--	128(Saraf et al., 2003)	DSC	15.4(Goebel et al., 2006)
87	AWAKIT	14435-92-8	--	--	--	400(Li et al., 2015)	--	--

88	AZCYHO	24824-15-5	--	--	--	--		--
89	BIZKOM01	1564257-34-6	--	--	--	124(Kettner et al., 2014)	DSC	--
90	CAZCEN	125363-08-8	--	--	--	--	--	--
91	CIHQIT	155438-10-1				--	--	56(Qu et al., 2018)
92	CUGDIR	99393-63-2	--	--	--	200(Zhang et al., 2002)	DSC	>200(Wikipedia, 2006)
93	DIXDET	268748-97-6	--	--	--	--	--	--
94	DIXFEV	137538-62-6	--	--	--	--	--	--
95	EJEGIJ	155438-13-4	--	--	--	300(Sinditskii et al., 2016)	--	--
96	EJEGOP	260963-78-8	--	--	--	--	--	--
97	EJEGUV	155438-14-5	--	--	--	--	--	70(Sheremetev et al., 1998)
98	EJEHAC	612518-65-7	--	--	--	--	--	147(Averkiev et al., 2003)
99	FEPVON	1415050-06-4	--	--	--	176(Chavez et al., 2012)	DSC	124(Chavez et al., 2012)
100	FORMOQ	1638095-71-2	--	--	--	140(Fischer et al., 2014)	DSC	--
101	GEPRAU	210626-81-6	--	--	--	--	--	--

102	LITSIQ	292856-78-1	--	--	--	--	--	--
103	NIYDUU	206446-59-5	--	--	--	149.9(10)(Liu et al., 2015)	DSC	97.4(Liu et al., 2015)
104	OXAYES	162111-36-6	--	--	--	212(Veauthier et al., 2010)	DSC	127(Veauthier et al., 2010)
105	QQQBRD02	918-37-6	689(●)(Meyer et al., 2007)	7.58(●)(Pepekin et al., 2011)	23.6(●)(Pepekin et al., 2011)	136.1(Huang et al., 2015)	DSC	150(Wikipedia, 2006)
106	RABSUE	157628-84-7	--	--	--	220(decomp)(Makhova et al., 2003)	--	220(decomp)(Makhova et al., 2003)
107	RABTAL	155438-27-0	--	--	--	--	--	--
108	RAVSOW	33406-97-2	--	--	--	--	--	--
109	REQYIW	174092-36-5	--	--	--	--	--	235(Sheremetev et al., 1996)
110	SEJHEU	162111-38-8	--	--	--	232.23(Li et al., 2009)	DSC	230(Li et al., 2009)
111	TIBKAQ	155438-28-1	--	--	--	--	--	--
112	TIBKEU	178043-06-6	--	--	--	--	--	--
113	TIZMAQ	152845-81-3	--	--	--	190(Sinditskii et al., 2016)	--	63(Sheremetev et al., 1998)
114	UBAWUR	371227-83-7	--	--	--	148(Leonard et al., 2011)	DSC	99(Leonard et al., 2011)

115	UHAMAR	152845-82-4	--	--	--	--	--	112(Sheremetev et al., 1998)
116	UHOYIB	155256-96-5	--	--	--	--	--	103(Sheremetev et al., 2015)
117	ZULDOZ	17557-81-2	--	--	--	--	--	40(Ulpiani, 1912)
118	HNOBEN	15834-75-0	1650(●)(Rice and Hare, 2002)	9.30(●)(Keshavarz, 2008)	42.1(●)(Dong and Zhou, 1989)	261.9(Zeman, 1980)	DTA	240(Nielsen et al., 1979)
119	FEYMEC	29306-57-8	--	--	--	--	--	131(Manelis et al., 2006)
120	BZOFOX	3470-17-5	1410(●)(Dong and Zhou, 1989)	8.26(●)(Keshavarz, 2008)	35.1(○)(Akst, 1989)	289(10)(Yang et al., 2012)	DSC	193(Ohta et al., 1963)
121	AFUGEP	782438-60-2	--	8.68(●)(Tian et al., 2011)	36.1(●)(Tian et al., 2011)	186.0(10)(Zhang et al., 2014)	DSC	82.6(Zhang et al., 2014)
122	XERPAM	371951-09-6	1383.6(●)(Zhou et al., 2011)	8.93(●)(Zhou et al., 2011)	--	272.0(5)(LI et al., 2016)	DSC	109.0(LI et al., 2016)
123	BADNAY01	1809272-88-5	--	--	--	138.0(Terrier et al., 1990)	--	--

124	SEFVOR	2195346-95-1	--	--	--	233.1(5)(Tang et al., 2017)	DSC	205.5(Tang et al., 2017)
125	PUBMII01	189192-28-7	--	--	--	> 195(decomp)(Meyer et al., 2016)	--	> 195(decomp)(Meyer et al., 2016)
126	DEDBUJ	98686-54-5	--	--	--	--	--	--
127	HIQBER	152678-74-5	--	--	--	243.0(5)(Kumar et al., 2018)	DSC	209.0(Kumar et al., 2018)
128	VETWAS	131394-26-8	--	--	--	290.0(5)(Liu et al., 2015)	DSC	--
129	DAZDUF	134293-22-4	--	--	--	214.0(5)(Fischer et al., 2012)	DSC	--
130	FIHPIY	2243211-28-9	--	--	--	228.0(5)(Tang et al., 2018)	DSC	--
131	KUBVIP	NA	--	--	--	340.8(5)(Li et al., 2019)	DSC	--
132	FOXHIM	131846-99-6	--	--	--	329(5)(Zhang et al., 2019)	DSC	--
133	OYAVIV	2095393-79-4	--	--	--	335.0(5)(Klapötke and Witkowski, 2016)	DSC	--
134	ZASWEX	2387677-24-7	--	--	--	--	--	--
135	ZASWAT	2387677-23-6	--	--	--	--	--	--

136	CUJFAQ	1801269-93-1	--	--	--	302.0(5)(Klapötke and Witkowski, 2016)	DSC	--
137	GEYQOT	NA	--	--	--	261.0(5)(Tang et al., 2018)	DSC	233.0(Tang et al., 2018)
138	MUKRAM	1198599-46-0	--	--	--	298.3(10)(Zeng et al., 2009)	DSC	
139	FOYSUJ	NA	--	--	--	197.9(Wu et al., 2015)	DSC	156.6(Wu et al., 2015)
140	CUDQUP	29754-26-5	--	--	--	260(10)(Hong et al., 2015)	DSC	67(10)(Hong et al., 2015)
141	GEXMON	1418127-35-1	--	--	--	--	--	171.3(10)(Zhang et al., 2013)
142	GEXMIH	1418127-36-2	--	--	--	--	--	205.8(10)(Zhang et al., 2013)
143	GEXMAZ	1418127-37-3	--	--	--	--	--	132.6(10)(Zhang et al., 2013)
144	GEXMED	1418127-38-4	--	--	--	--	--	189.0(10)(Zhang et al., 2013)
145	ZEVNUL	NA	--	--	--	--	--	164.5(10)(Zhang et al., 2013)
146	PEHSUS	2309306-47-4	--	--	--	235(10)(Yang et al., 2012)	DSC	220(Yang et al., 2012)
147	TOZMUS	NA	--	--	--	240(10)(Hong et al., 2015)	DSC	62(10)(Hong et al., 2015)

148	IZUZUZ	1583315-32-5	--	--	--	207(10)(Bolton et al., 2011)	DSC	136(10)(Bolton and Matzger, 2011)
149	TIVJUF	2387677-27-0	--	--	--	216.8(10)(Wang et al., 2014)	DSC	136.7(10)(Wang et al., 2014)
150	QISTAN01	250165-39-0	--	--	--	--	--	--
151	GOWHIL	NA	--	--	--	220(10)(Yang et al., 2014)	DSC	91(10)(Yang et al., 2014)
152	ZEBJOH	1668570-32-8	--	--	--	243.5(10)(Gao et al., 2014)	DSC	-
153	FIHPEU	2243696-54-8	--	--	--	315.1(5)(Tang et al., 2018)	DSC	--

Table S4. Feature importance ranking by the magnitude of Pearson correlation coefficients. (Related to Figure 5)

**Label**	**Feature**	***Importance to Q_{max}***	**Feature**	***Importance to D***	**Feature**	***Importance to $p_{C\text{-}J}$***	**Feature**	***Importance to T_d***	**Feature**	***Importance to LE***
1	Product gaseous CO_2	0.610	Product solid C	-0.813	Product solid C	-0.820	Oxygen balance	-0.430	Incrystal mix with hydrogen-rich molecules	0.698
2	HB strength	-0.597	Oxygen balance	0.806	Oxygen balance	0.803	HB amount	0.390	HB strength	0.603
3	Functional group -NH_2	-0.472	Material density	0.748	Material density	0.794	Weakest bond strength	0.378	HB amount	0.564
4	Product gaseous H_2O	-0.378	Product gaseous CO_2	0.691	Product gaseous CO_2	0.700	HB length	0.287	Incrystal mix with energetic molecules	0.536
5	Product gaseous O_2	-0.367	Product gaseous N_2	0.544	Product gaseous N_2	0.549	Molecular weight	0.227	HB length	0.308
6	HB amount	-0.324	Crystal packing coefficient	0.362	Crystal packing coefficient	0.407	Number of molecules in a	-0.219	Material density	-0.288

							primitive cell			
7	Crystal packing type	0.307	Nitrogen density	0.290	Nitrogen density	0.301	Material density	-0.217	Molecular weight	0.274
8	Product gaseous NH_3	-0.300	Product gaseous NH_3	-0.281	HB amount	-0.265	Crystal packing type	-0.194	Oxygen balance	-0.272
9	HB length	-0.267	HB amount	-0.260	Functional group $-NO_2$	0.260	Functional group $-NH_2$	0.191	Weakest bond strength	0.245
10	Molecular weight	0.251	Incrystal mix with energetic molecules	-0.259	Incrystal mix with energetic molecules	-0.254	HB strength	0.190	Weakest bond type	-0.198
11	Oxygen balance	0.201	HB strength	-0.243	Product gaseous NH_3	-0.254	Weakest bond length	0.189	Molecular backbone	0.183
12	Product gaseous N_2	0.193	Functional group $-NO_2$	0.238	HB strength	-0.226	Functional group $-N_3$	0.185	Functional group $-NH_2$	0.148

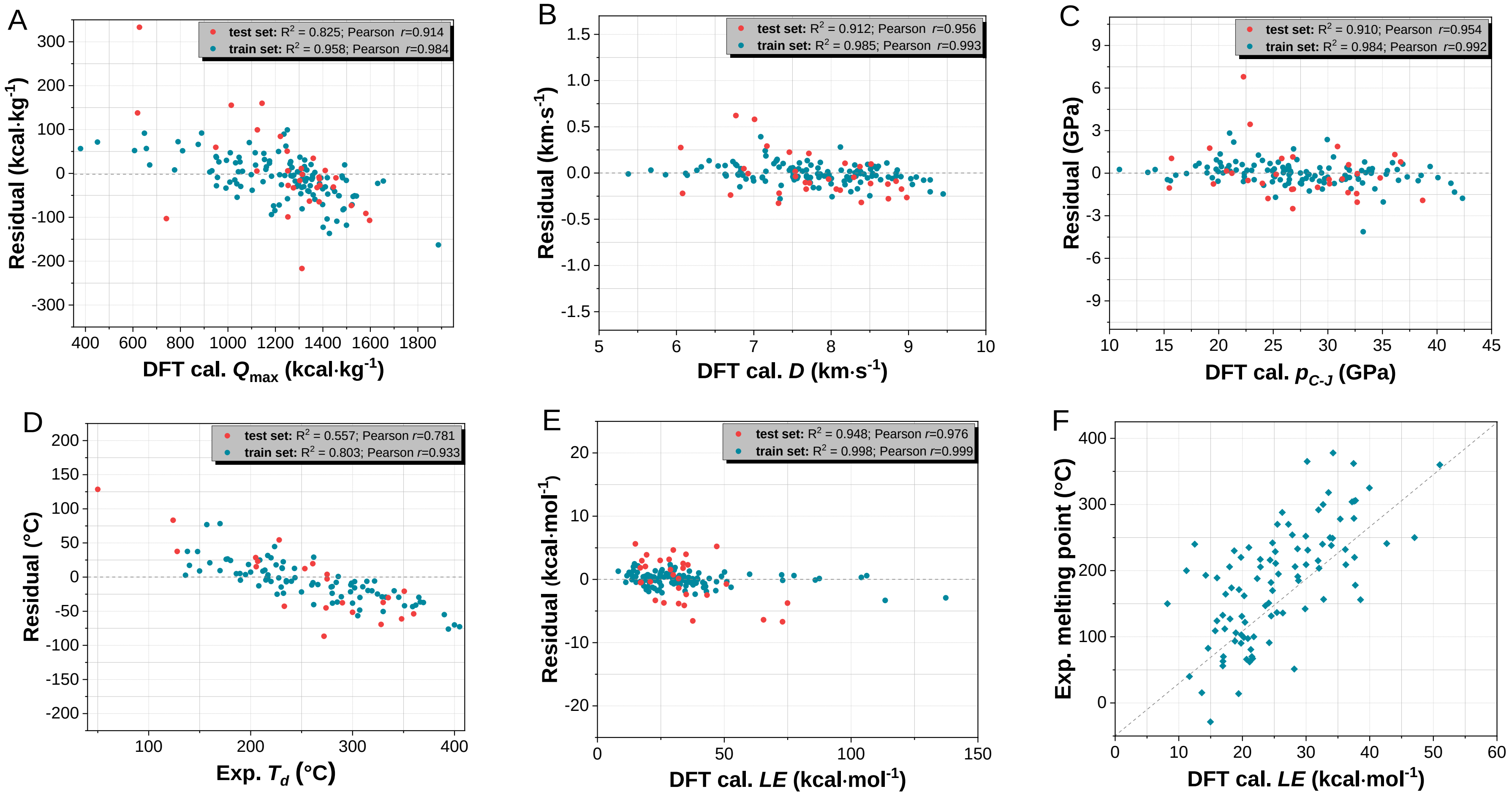

Figure S1. XGBoost prediction residuals for (A) Q_{max}, (B) D, (C) $p_{C\text{-}J}$, (D) T_d, and (E) *LE*. (F) Roughly positive correlation of melting temperature to *LE*. (Related to Table 1 and Figure 4)

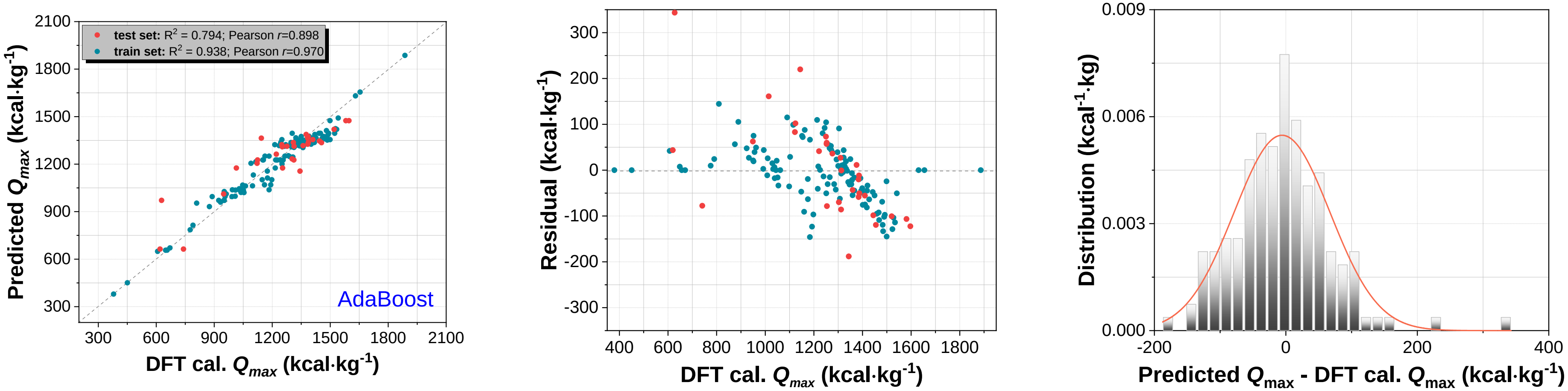

Figure S2. Prediction of heat of explosion with AdaBoost model. (Related to Table 1)

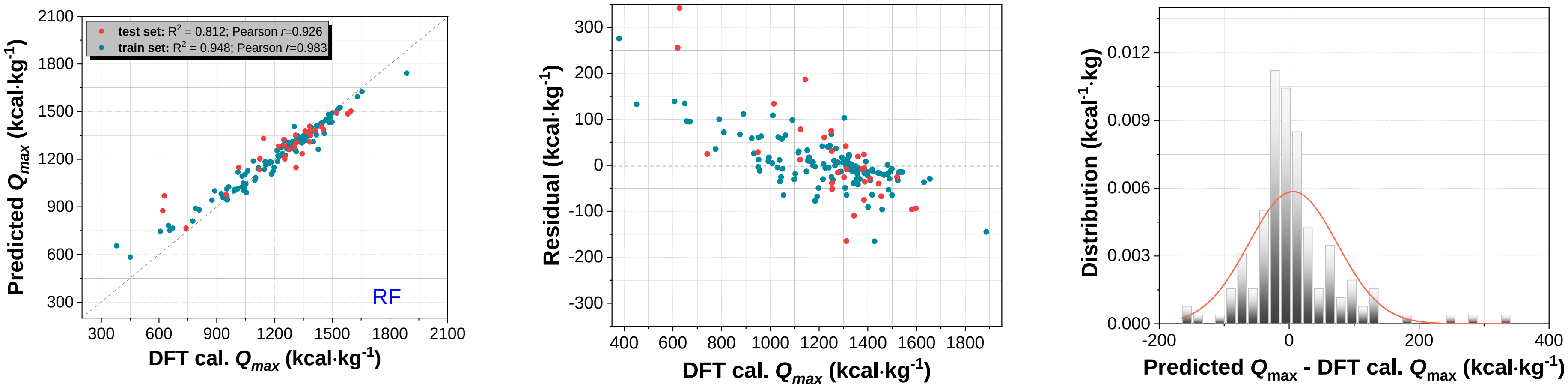

Figure S3. Prediction of heat of explosion with RF model. (Related to Table 1)

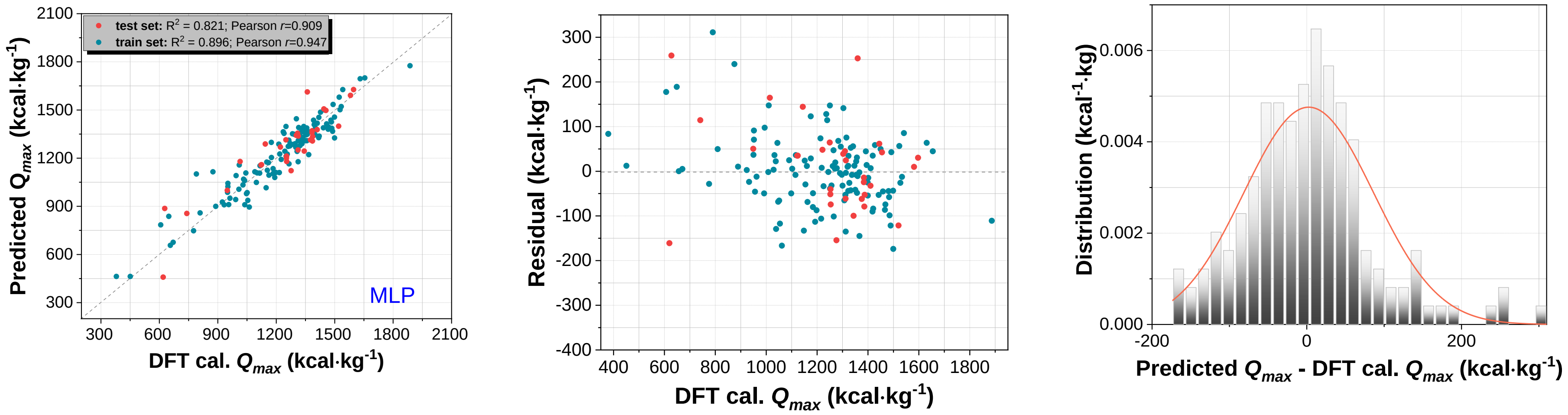

Figure S4. Prediction of heat of explosion with MLP model. (Related to Table 1)

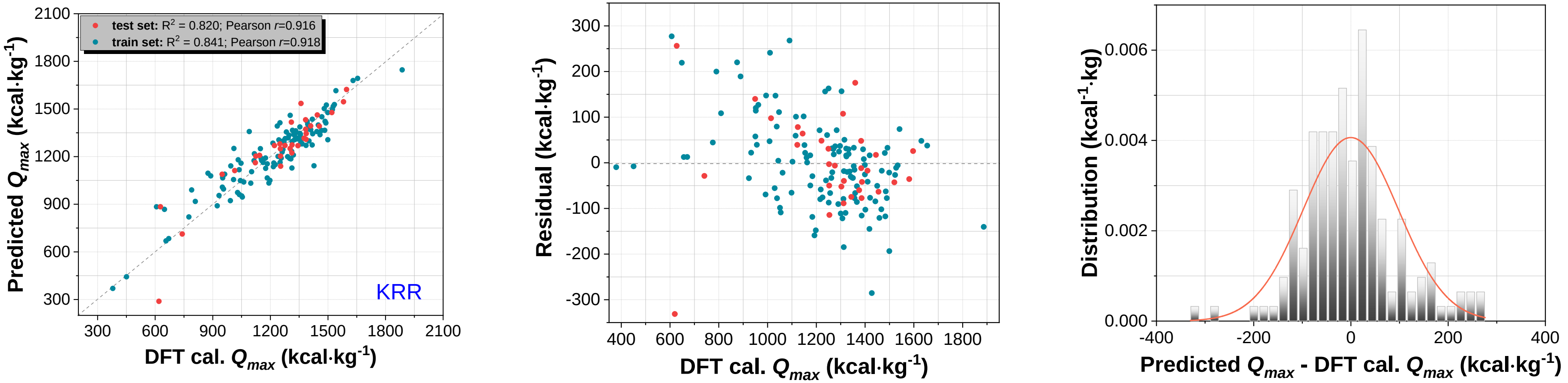

Figure S5. Prediction of heat of explosion with KRR model. (Related to Table 1)

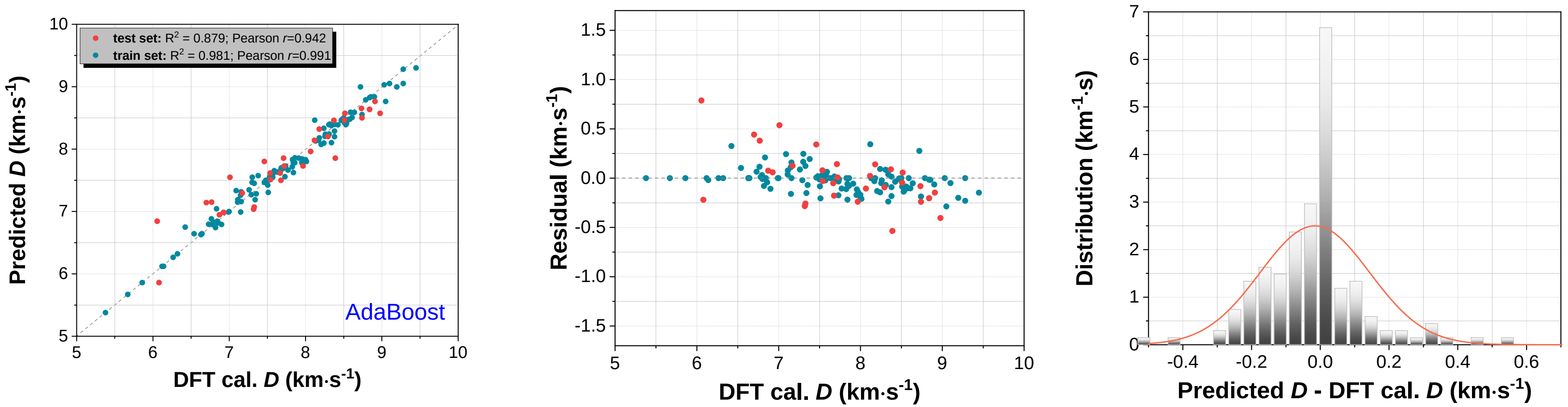

Figure S6. Prediction of detonation velocity with AdaBoost model. (Related to Table 1)

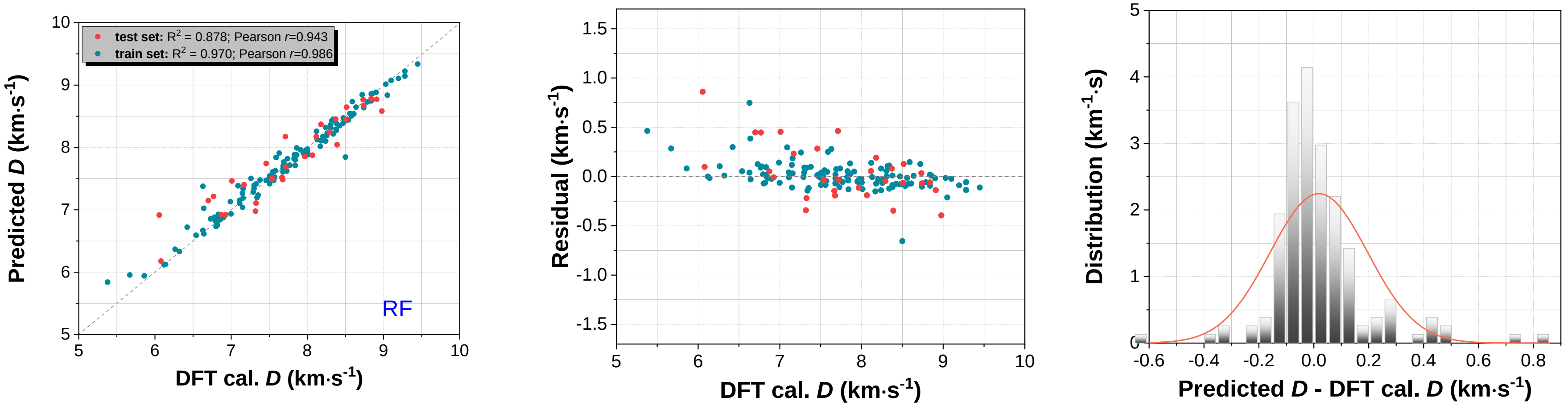

Figure S7. Prediction of detonation velocity with RF model. (Related to Table 1)

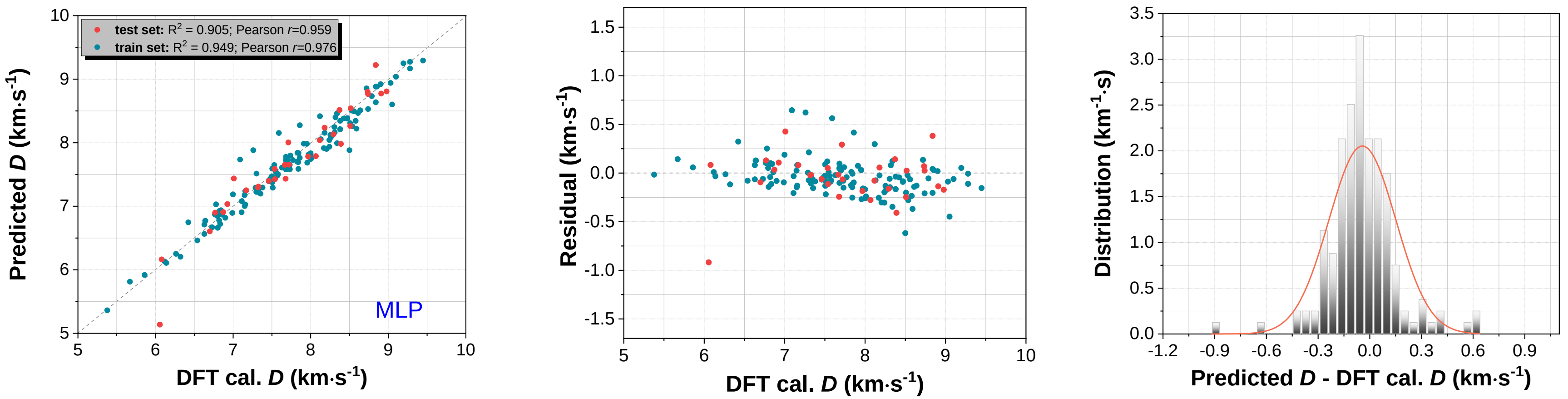

Figure S8. Prediction of detonation velocity with MLP model. (Related to Table 1)

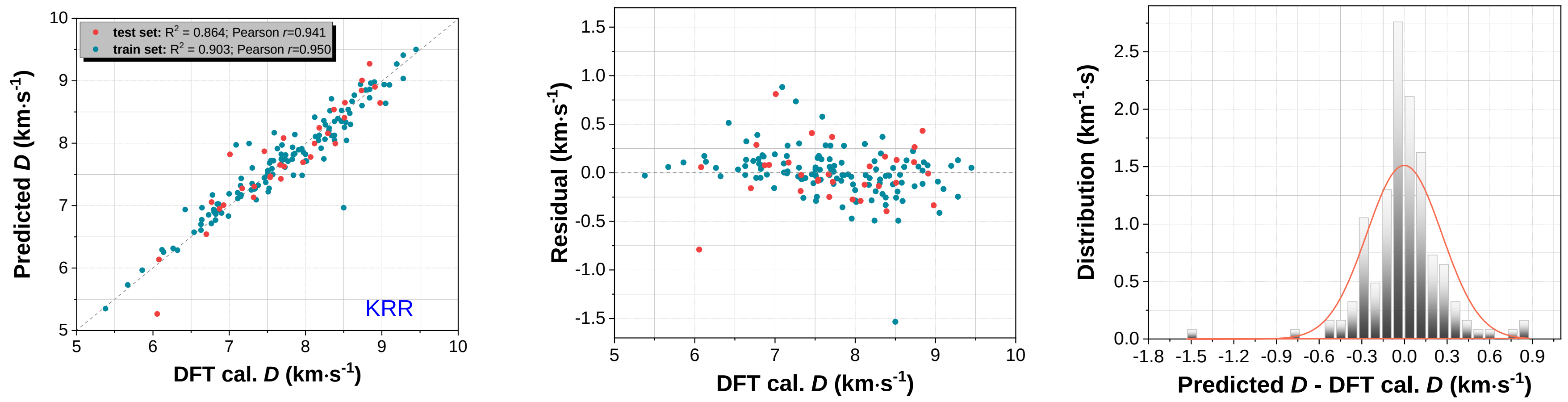

Figure S9. Prediction of detonation velocity with KRR model. (Related to Table 1)

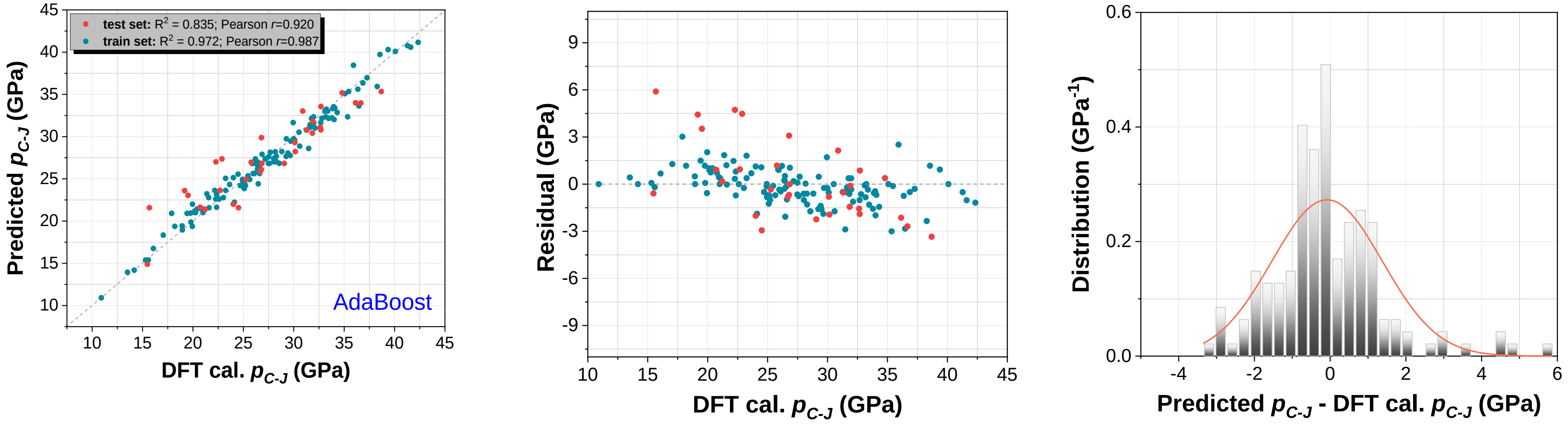

Figure S10. Prediction of detonation pressure with AdaBoost model. (Related to Table 1)

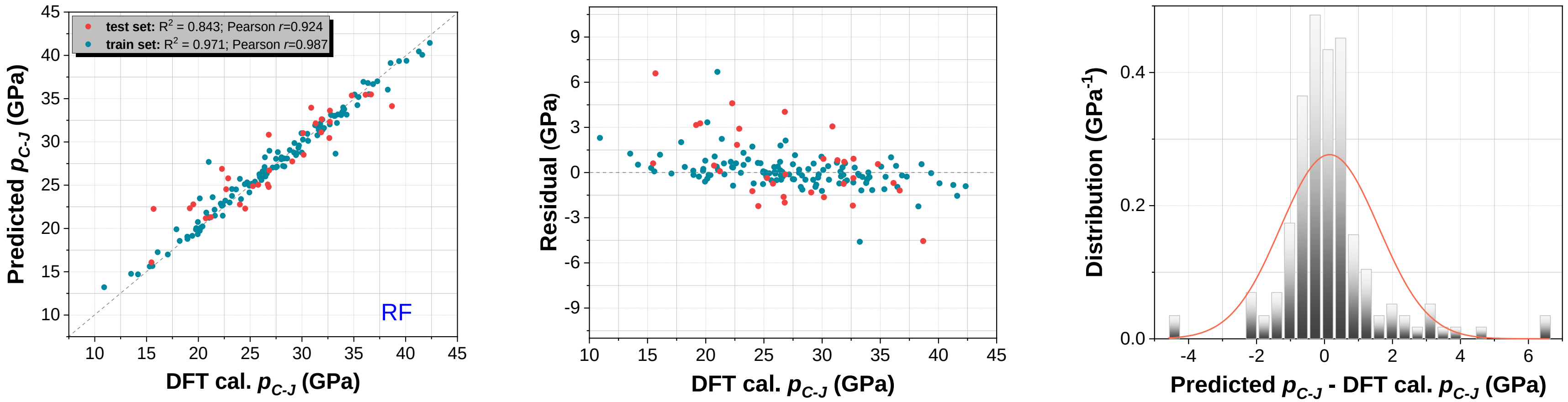

Figure S11. Prediction of detonation pressure with RF model. (Related to Table 1)

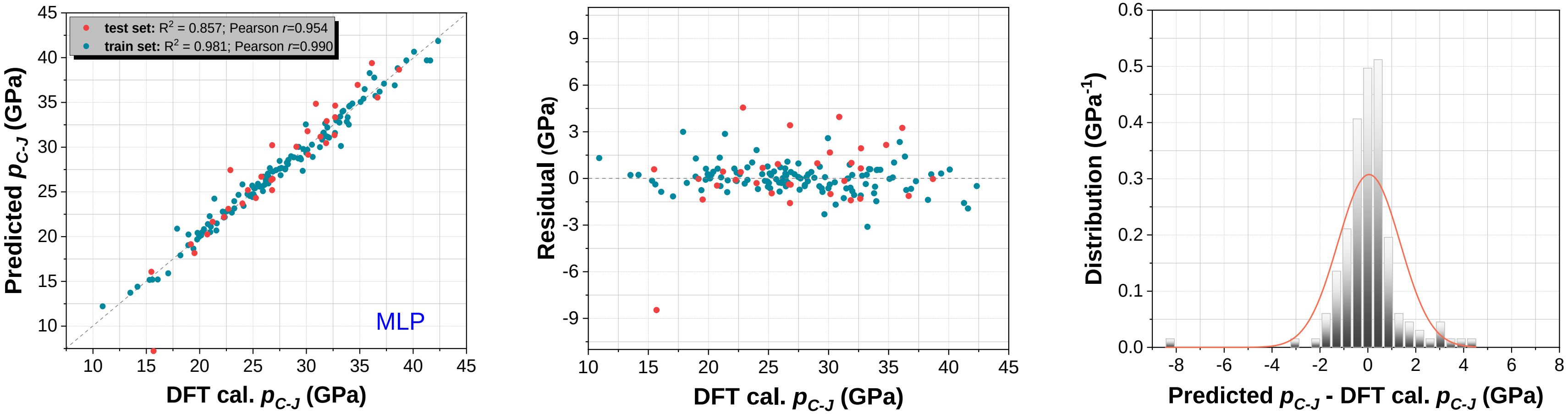

Figure S12. Prediction of detonation pressure with MLP model. (Related to Table 1)

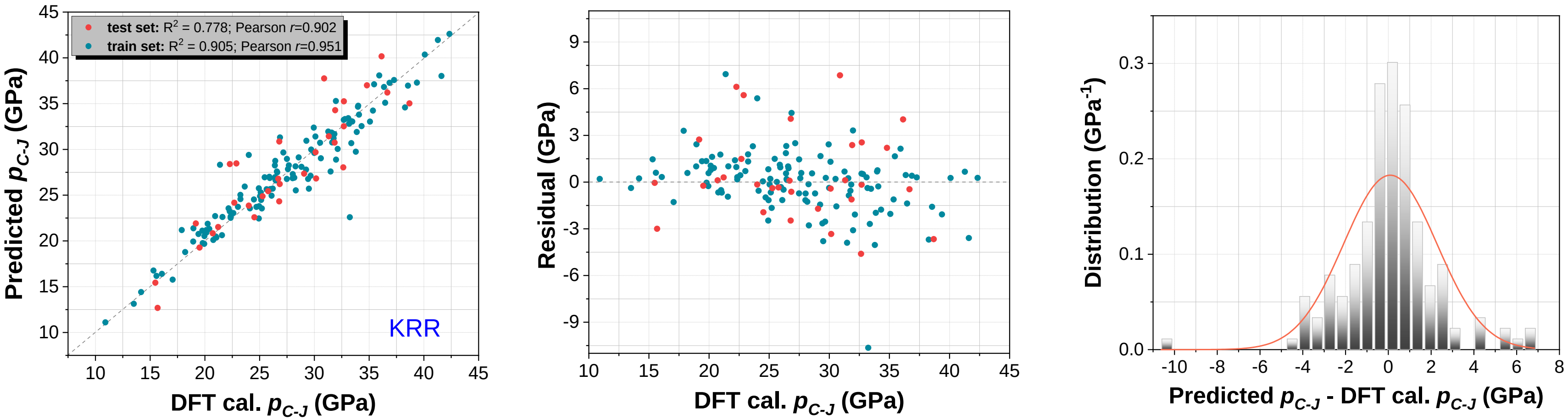

Figure S13. Prediction of detonation pressure with KRR model. (Related to Table 1)

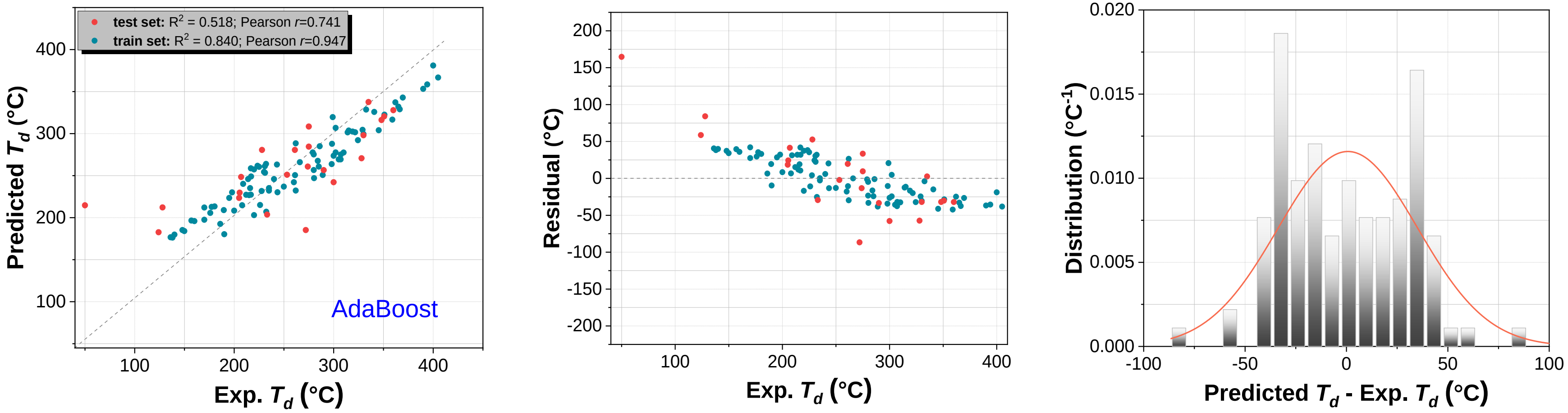

Figure S14. Prediction of decomposition temperature with AdaBoost model. (Related to Table 1)

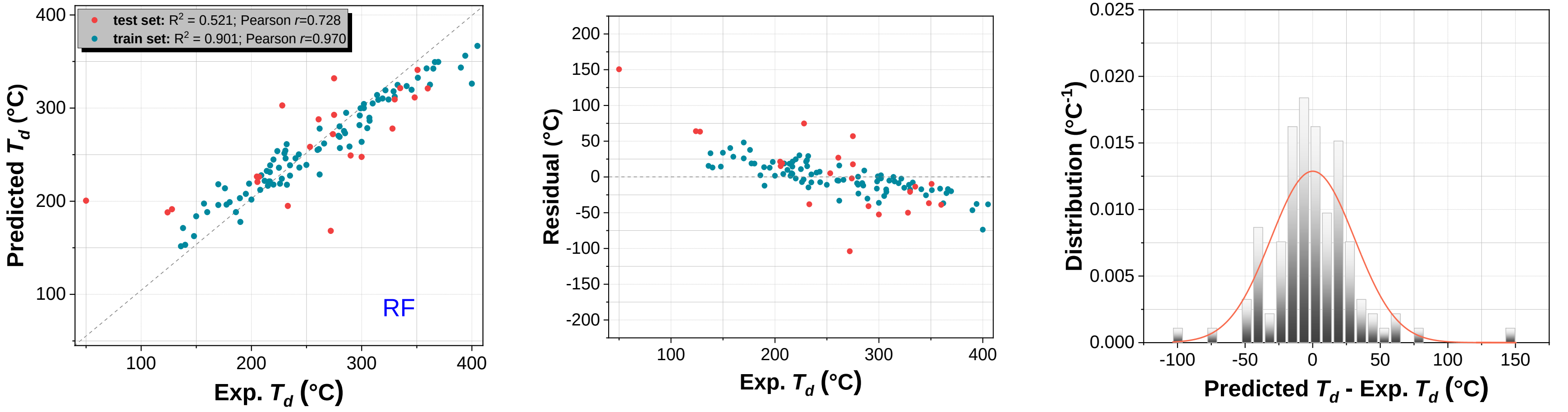

Figure S15. Prediction of decomposition temperature with RF model. (Related to Table 1)

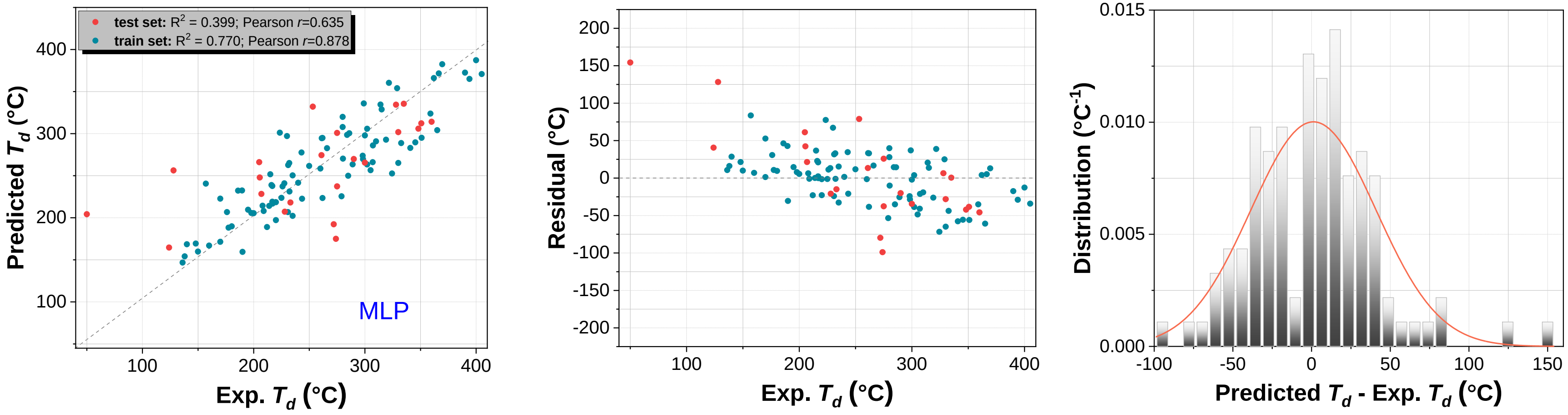

Figure S16. Prediction of decomposition temperature with MLP model. (Related to Table 1)

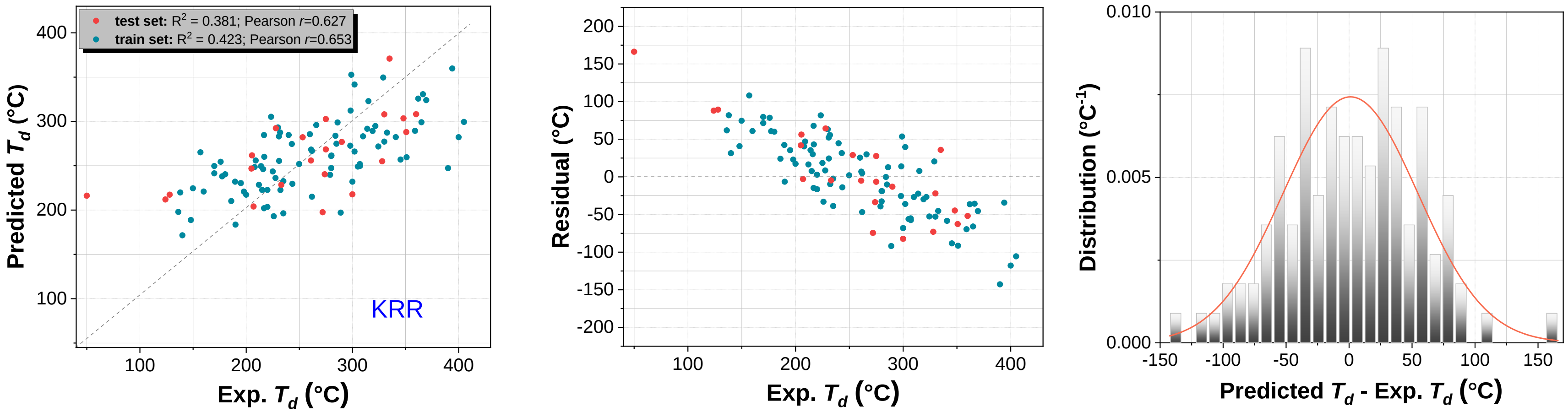

Figure S17. Prediction of decomposition temperature with KRR model. (Related to Table 1)

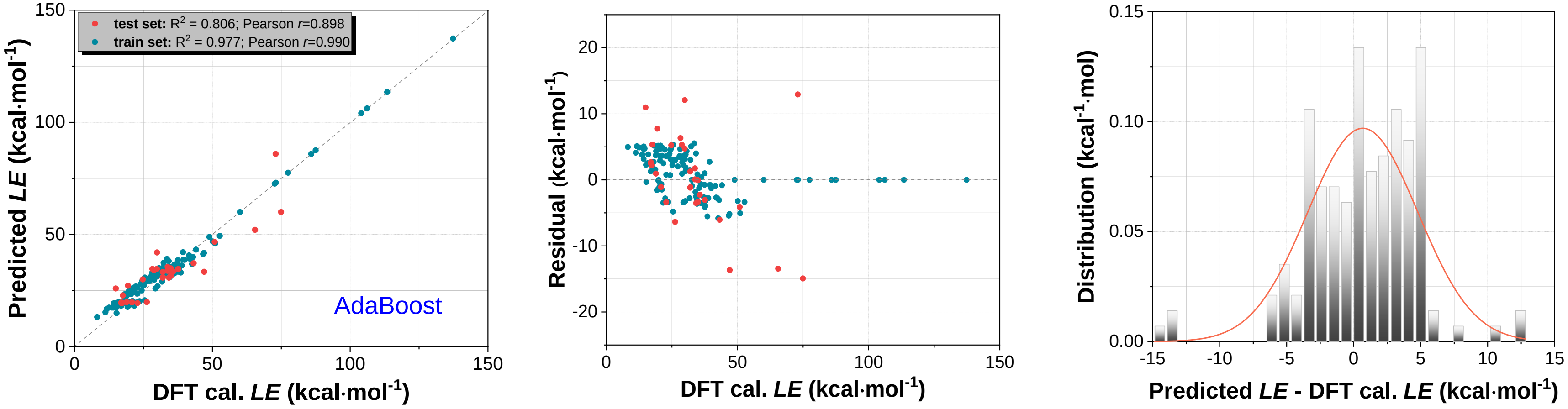

Figure S18. Prediction of lattice energy with AdaBoost model. (Related to Table 1)

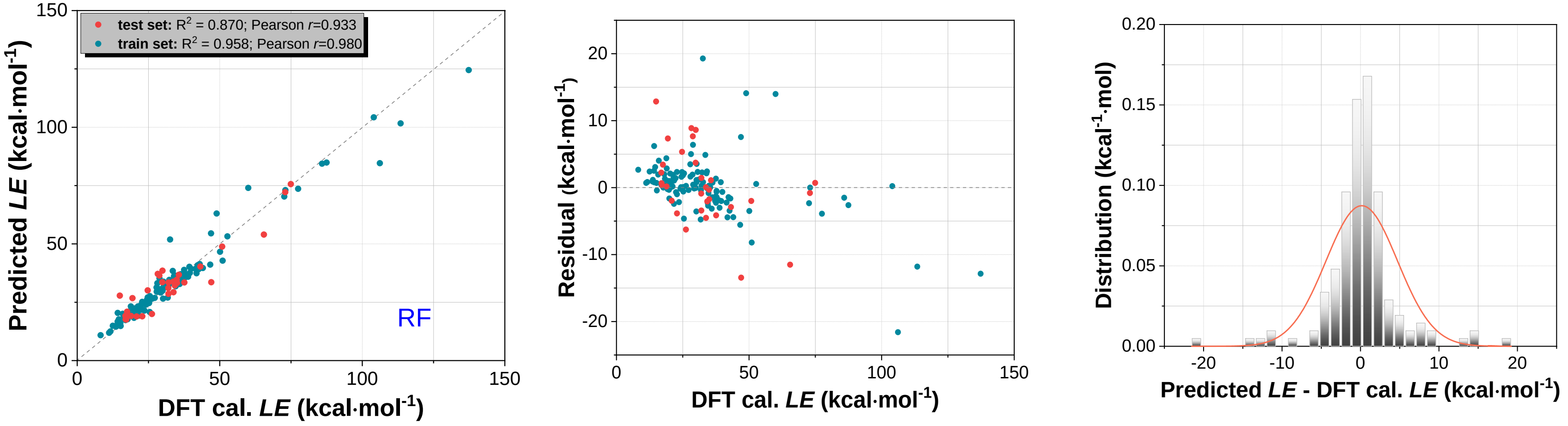

Figure S19. Prediction of lattice energy with RF model. (Related to Table 1)

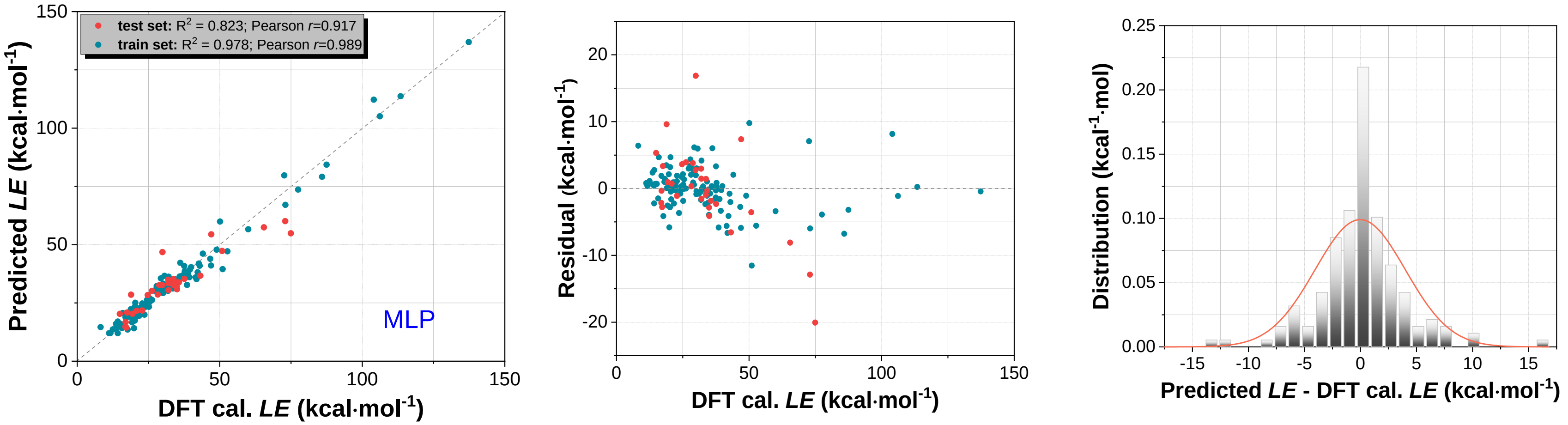

Figure S20. Prediction of lattice energy with MLP model. (Related to Table 1)

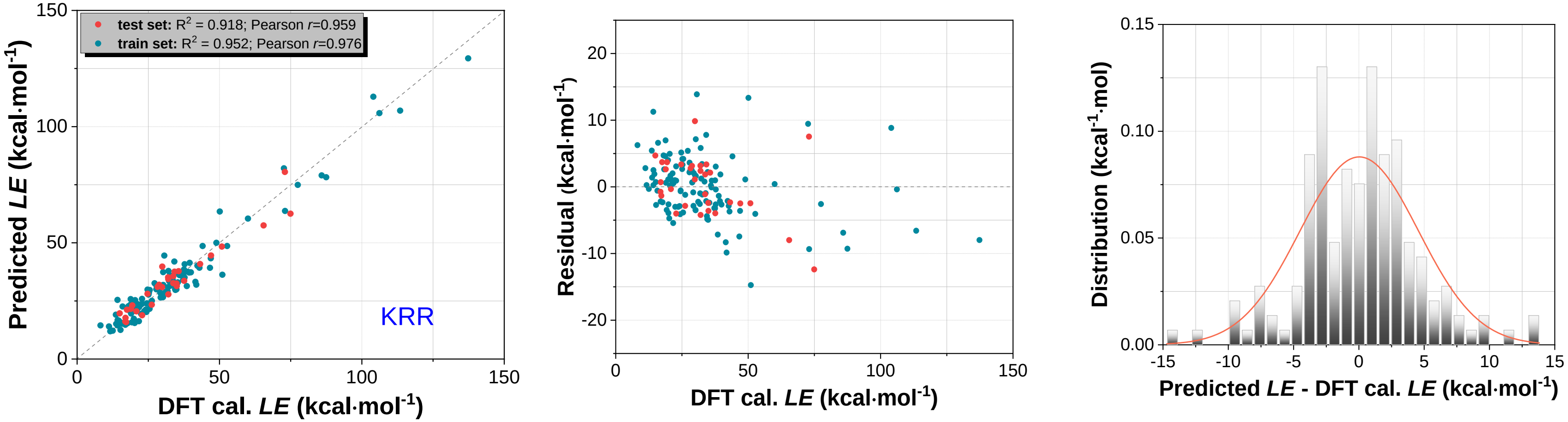

Figure S21. Prediction of lattice energy with KRR model. (Related to Table 1)

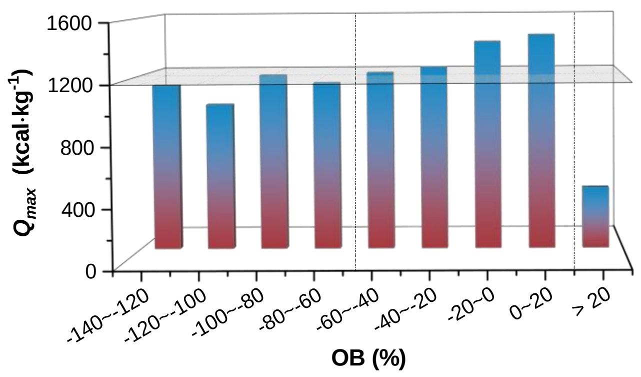

-60% < OB < 20%

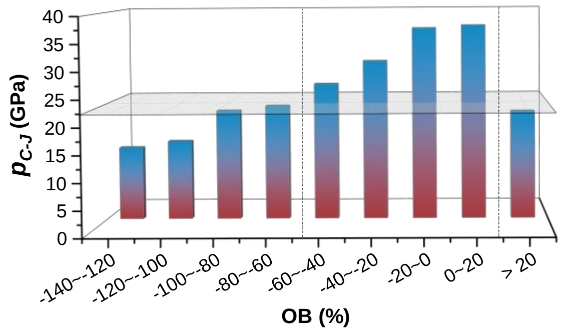

-60% < OB < 20%

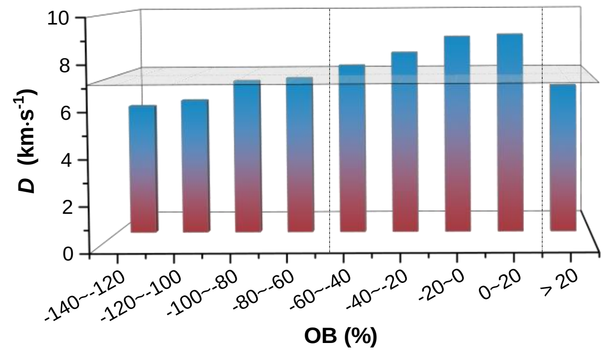

-60% < OB < 20%

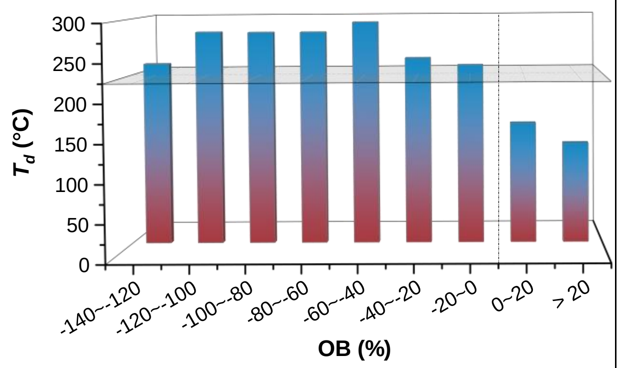

-140% < OB < 0%

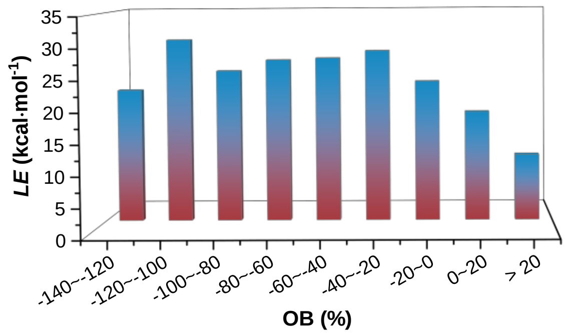

Weak relevance & Arbitrary range

Optimal range:

-60 < OB < 0%

Figure S22. Optimal range of oxygen balance in balancing detonation performance and stability of HEMDs. (Related to Table 2)

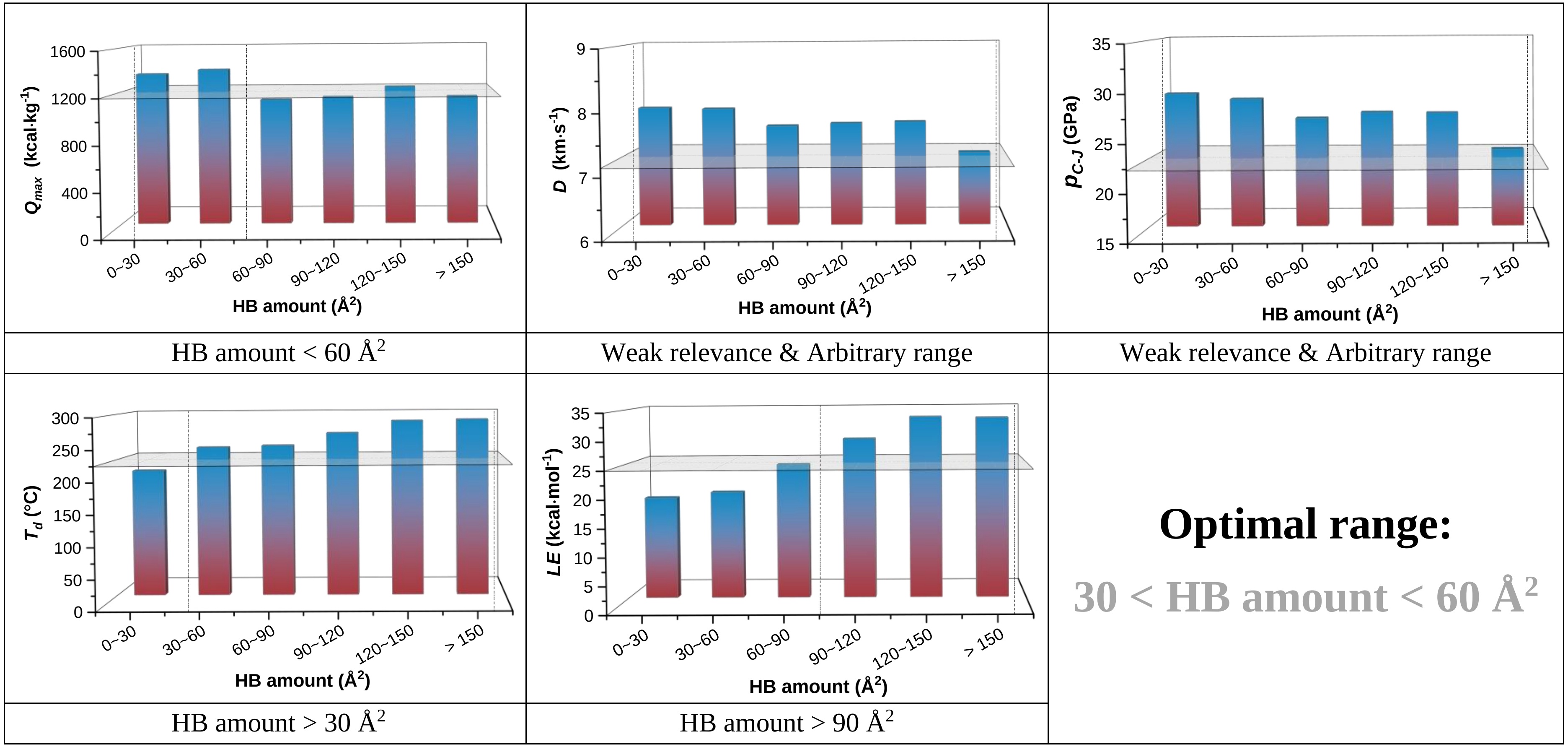

Figure S23. Optimal range of HB amount in balancing detonation performance and stability of HEMDs. (Related to Table 2)

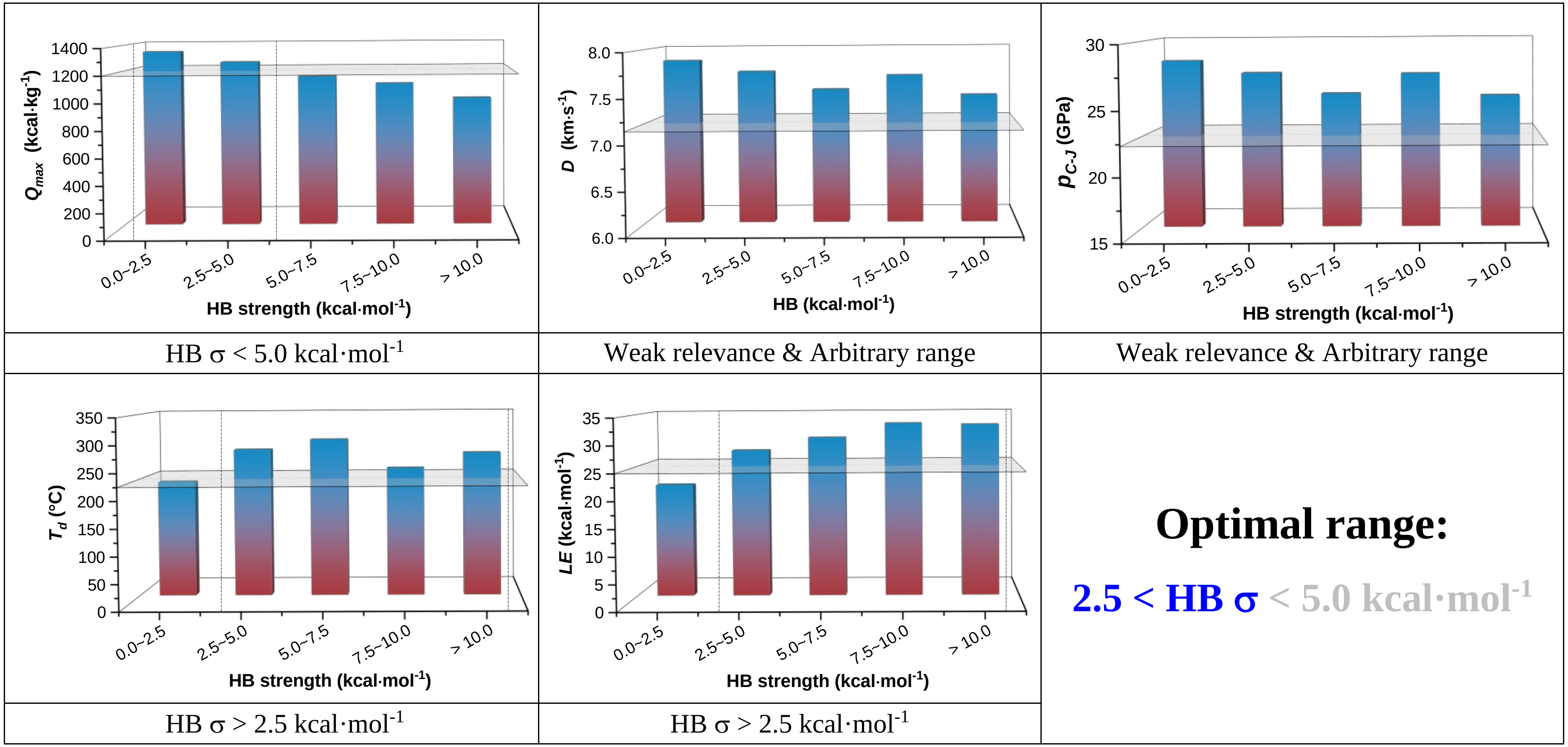

Figure S24. Optimal range of HB strength in balancing detonation performance and stability of HEMDs. (Related to Table 2)

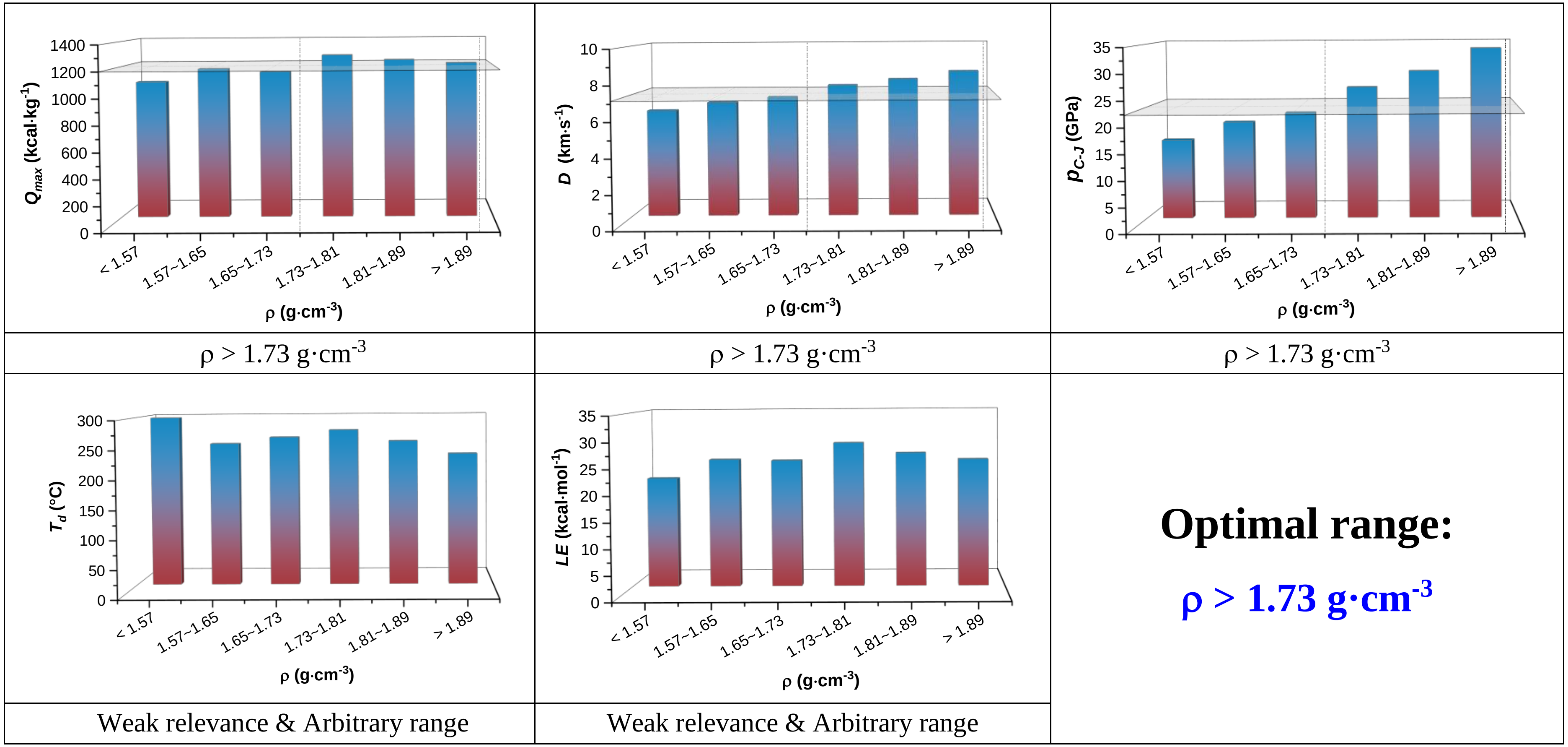

Figure S25. Optimal range of material density in balancing detonation performance and stability of HEMDs. (Related to Table 2)

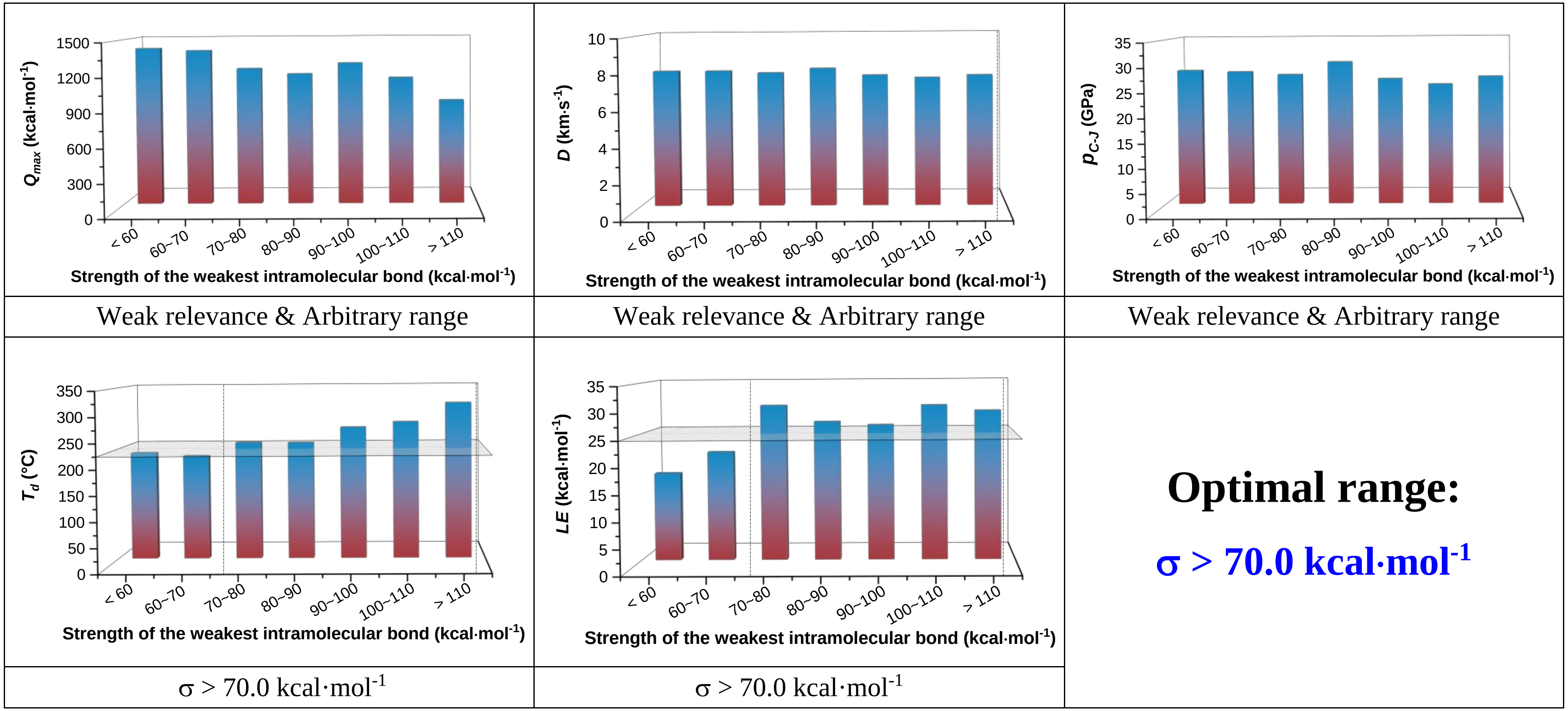

Figure S26. Optimal range of strength of the weakest intramolecular bond in balancing detonation performance and stability of HEMDs. (Related to Table 2)

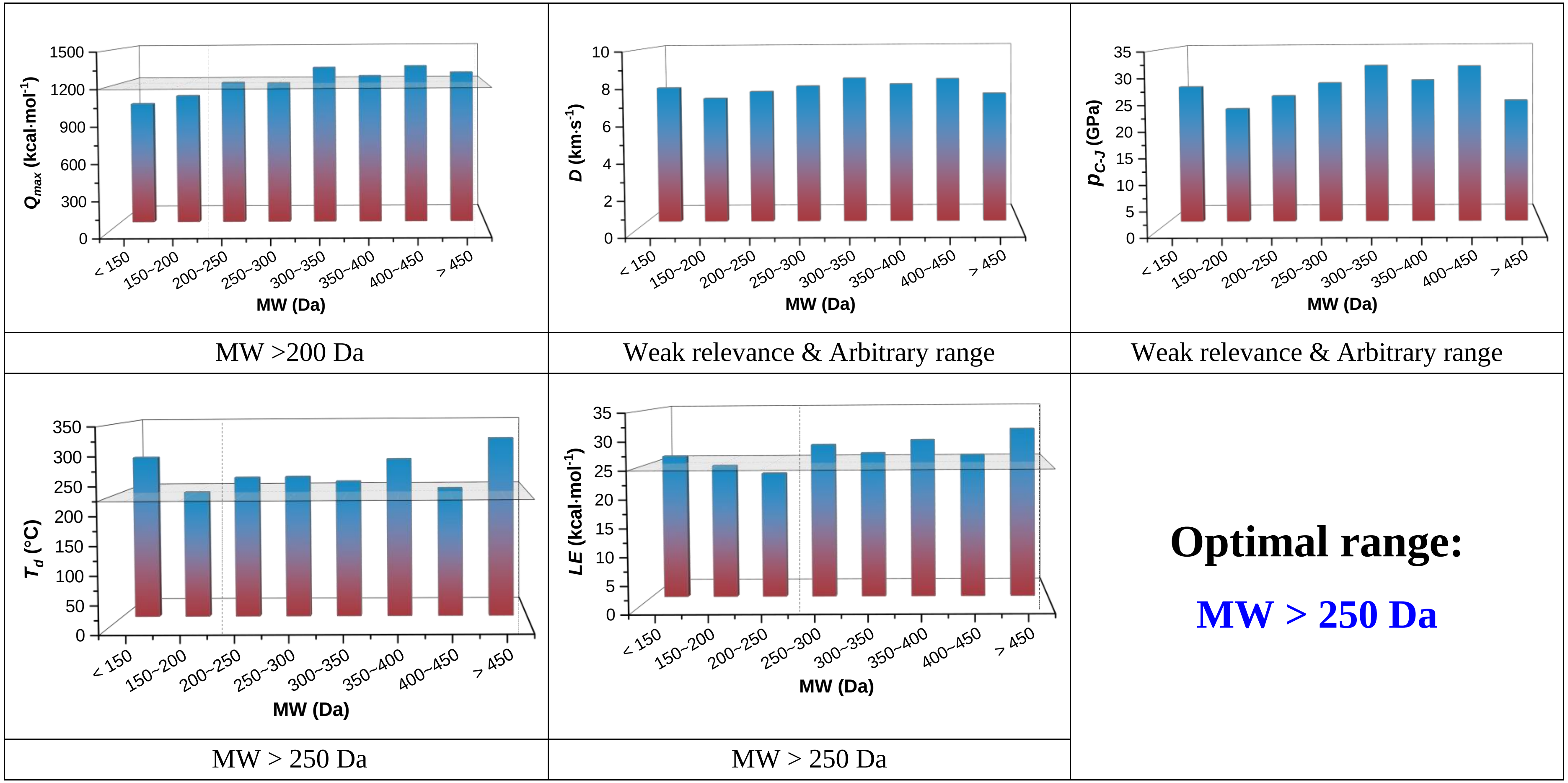

Figure S27. Optimal range of molecular weight in balancing detonation performance and stability of HEMDs. (Related to Table 2)

References

Akst, I. (1989). Heat of detonation, the cylinder test, and performance munitions. Los Alamos National Lab., NM (USA).

Altenburg, T., Klapötke, T.M., and Penger, A. (2009). Primary nitramines related to nitroglycerine: 1-nitramino-2, 3-dinitroxypropane and 1, 2, 3-trinitraminopropane. Cent. Eur. J. Energ. Mater. *6*, 255-275.

Altmann, K.L., Chafin, A.P., Merwin, L.H., Wilson, W.S., and Gilardi, R. (1998). Chemistry of tetraazapentalenes. J. Org. Chem. *63*, 3352-3356.

Antonangeli, D., Farber, D.L., Said, A.H., Benedetti, L.R., Aracne, C.M., Landa, A., Söderlind, P., and Klepeis, J.E. (2010). Shear softening in tantalum at megabar pressures. Phys. Rev. B *82*, 132101.

Atkins, R.L., Hollins, R.A., and Wilson, W.S. (1986). Synthesis of polynitro compounds. Hexasubstituted benzenes. J. Org. Chem. *51*, 3261-3266.

Averkiev, B., Antipin, M.Y., Yudin, I., and Sheremetev, A. (2002). X-ray structural study of three derivatives of dinitropyrazine. J. Mol. Struct. *606*, 139-146.

Averkiev, B.B., Antipin, M.Y., Sheremetev, A.B., and Timofeeva, T.V. (2003). Four 3-cyanodifurazanyl ethers: potential propellants. Acta Crystallogr. Sect. C: Cryst. Struct. Commun. *59*, o383-o387.

Bachman, G.B., and Vogt, C.M. (1958). The BF3· N2O4 complex as a nitrating agent1. J. Am. Chem. Soc. *80*, 2987-2991.

Bagryanskaya, I.Y., and Gatilov, Y.V. (1983). Crystal structure of nitromethane. J. Struct. Chem. *24*, 150-151.

Bemm, U., and Östmark, H. (1998). 1,1-diamino-2,2-dinitroethylene: A novel energetic material with infinite layers in two dimensions. Acta Crystal. *54*, 1997–1999.

Blanksma, J.J. (1908). Bromination and Nitration of Meta-Substituted Phenols. Rec. Trav. Chim. *27*, 25-41.

Boileau, J., Carail, M., Wimmer, E., Gallo, R., and Pierrot, M. (1985). Dérivés nitrés acétylés du glycolurile. Propellants, Explos., Pyrotech. *10*, 118-120.

Bölter, M.F., Klapötke, T.M., Kustermann, T., Lenz, T., and Stierstorfer, J. (2018). Improving the Energetic Properties of Dinitropyrazoles by Utilization of Current Concepts. Eur. J. Inorg. Chem. *2018*, 4125-4132.

Bolton, O., and Matzger, A.J. (2011). Improved stability and smart-material functionality realized in an energetic cocrystal. Angew. Chem. *123*, 9122-9125.

Boyer, J., and Morgan, J., L (1959). Acid catalyzed reactions between carbonyl compounds and organic azides. II. aromatic aldehydes. J. Org. Chem. *24*, 561-562.

Cai, H., Shu, Y., Huang, H., Cheng, B., and Li, J. (2004). Study on reactions of 2-(dinitromethylene)-4, 5-imidazolidinedione. J. Org. Chem. *69*, 4369-4374.

Chavez, D.E., Parrish, D.A., and Leonard, P. (2012). The synthesis and characterization of a new furazan heterocyclic system. Synlett *23*, 2126-2128.

Chaykovsky, M., and Adolph, H.G. (1990). Synthesis and properties of some trisubstituted trinitrobenzenes. TATB analogs. J. Energ. Mater. *8*, 392-414.

Colonna, G.R., and Spencer, A.B. (2010). Fire Protection Guide to Hazardous Materials (14th edition) (National Fire Protection Association).

Crawford, M.J., Evers, J., Göbel, M., Klapötke, T.M., Mayer, P., Oehlinger, G., and Welch, J.M. (2007).

γ - FOX - 7: Structure of a High Energy Density Material Immediately Prior to Decomposition. Propellants, Explos., Pyrotech. *32*, 478-495.
Davis, T.L., and Abrams, A.J. (1925). The Dehydration of Ammonium Nitrate. J. Am. Chem. Soc. *47*, 1043-1045.
Dobratz, B.M., and Crawford, P.C. (1985). LLNL explosives handbook - properties of chemical explosives and explosive simulants (Lawrence Livemore National Laboratory).
Domasevitch, K.V., Gospodinov, I., Krautscheid, H., Klapötke, T.M., and Stierstorfer, J. (2019). Facile and selective polynitrations at the 4-pyrazolyl dual backbone: straightforward access to a series of high-density energetic materials. New J. Chem. *43*, 1305-1312.
Dong, H., and Zhou, F. (1989). High-Energy Explosives and Related Properties (Science Press).
Du, Y., Wang, Y.H., Li, Y.J., Zheng, Z.H., and Wang, J.L. (2013). Theoretical Calculation of the Detonation Parameters of ANPyO and Comparison between ANPyO and me (ANPyO) x on their Performance. Adv. Mater. Res. *791*, 60-63.
Fischer, D., Gottfried, J.L., Klapötke, T.M., Karaghiosoff, K., Stierstorfer, J., and Witkowski, T.G. (2016). Synthesis and investigation of advanced energetic materials based on bispyrazolylmethanes. Angew. Chem. *128*, 16366-16369.
Fischer, D., Klapötke, T.M., and Stierstorfer, J. (2014). Synthesis and characterization of diaminobisfuroxane. Eur. J. Inorg. Chem. *2014*, 5808-5811.
Fischer, N., Fischer, D., Klapötke, T.M., Piercey, D.G., and Stierstorfer, J. (2012). Pushing the limits of energetic materials–the synthesis and characterization of dihydroxylammonium 5, 5′-bistetrazole-1, 1′-diolate. J. Mater. Chem. *22*, 20418-20422.
Fonger, G.C., Hakkinen, P., Jordan, S., and Publicker, S. (2014). The National Library of Medicine's (NLM) Hazardous Substances Data Bank (HSDB): background, recent enhancements and future plans. Toxicology *325*, 209-216.
Fried, L.E. (1998). LLNL CHEETAH Reactant Library. 1.0 ed.: SANDIA REPORT SAND98-1191, Unlimited Release.
Gao, B., Wang, D., Zhang, J., Hu, Y., Shen, J., Wang, J., Huang, B., Qiao, Z., Huang, H., Nie, F., et al. (2014). Facile, continuous and large-scale synthesis of CL-20/HMX nano co-crystals with high-performance by ultrasonic spray-assisted electrostatic adsorption method. J. Mater. Chem. A *2*, 19969-19974.
Gill, R., Asaoka, L., and Baroody, E. (2006). On underwater detonations, 1. A new method for predicting the CJ detonation pressure of explosives. J. Energ. Mater. *5*, 287-307.
Goebel, M., Klapoetke, T.M., and Mayer, P. (2006). Crystal structures of the potassium and silver salts of nitroform. Z. Anorg. Allg. Chem. *632*, 1043-1050.
Gore, G., Sivabalan, R., Nair, U., Saikia, A., Venugopalan, S., and Gandhe, B. (2007). Synthesis of CL-20: By oxidative debenzylation with cerium (IV) ammonium nitrate (CAN). Indian J. Chem. *46B*, 505-508.
Guillou, S., Jacob, G., Terrier, F., and Goumont, R. (2009). An unexpected synthesis of 7-azidofurazano[3,4-b]tetrazolopyrazine. Tetrahedron *65*, 8891-8895.
Guo, J., Zhang, T., Zhang, J., and Liu, Y. (2006). Experimental Studies on the 2,4,6-Trinitro-m-Xylene Crystal. Chin. J. Explos. Propellants *29*, 58-62.

Hall, R.H., and Wright, G.F. (1951). Reaction of acetyl chloride with 1-nitro-2-nitramino-2-propoxyimidazolidine. J. Am. Chem. Soc. *73*, 2213-2216.

He, Z., Yan, S., and Liu, Z. (2013). Thermal Decomposition Characteristics of 2,6-Diamino-3,5-dinitropyridine-1-oxide. Chin. J. Explos. Propellants *36*, 51.

Headqurters, D.T.A. (1984). Military Explosives (Headqurters department the army).

Hobbs, M.L., and Baer, M.R. (1993) Published. Calibrating the BKW-EOS with a large product species data base and measured C-J properties. 10th Symposium (International) on Detonation, 1993 of Conference Boston, Massachusetts. 409.

Hong, D., Li, Y., Zhu, S., Zhang, L., and Pang, C. (2015). Three insensitive energetic co-crystals of 1-nitronaphthalene, with 2, 4, 6-trinitrotoluene (TNT), 2, 4, 6-trinitrophenol (picric acid) and D-mannitol hexanitrate (MHN). Cent. Eur. J. Energ. Mater. *12*, 47-62.

Huang, H., Shi, Y., Yang, J., and Li, B. (2015). Compatibility study of dihydroxylammonium 5, 5′-bistetrazole-1, 1′-diolate (TKX-50) with some energetic materials and inert materials. J. Energ. Mater. *33*, 66-72.

Huang, H., Zhou, Z., Song, J., Liang, L., Wang, K., Cao, D., Sun, W., Dong, X., and Xue, M. (2011). Energetic salts based on dipicrylamine and its amino derivative. Chemistry–A European Journal *17*, 13593-13602.

Huang, J., Cheng, B., Ma, Q., and Nie, F. (2011). The study of synthesis technology and performance of HNBB. Chin. J. Energ. Mater. *19*, 240.

Huang, Q., Ma, Y., Guo, Z., Liao, L., Hao, S., Nie, F., and Li, H. (2019). An unexpected method to synthesize fluorinated derivative of ANPZ. Propellants, Explos., Pyrotech. *44*, 1521-1527.

Hussein, A.K., Zeman, S., and Elbeih, A. (2018). Synthesis, Performance, and Thermal Behavior of a Novel Insensitive EDNA/DAT Co-crystal. Zeitschrift für anorganische und allgemeine Chemie *644*, 430-437.

Jiao, Q., Zhu, Y., Xing, J., Ren, H., and Huang, H. (2014). Thermal Decomposition of Rdx/Ap by Tg–Dsc–Ms–Ftir. J. Therm. Anal. Calorim. *116*, 1125-1131.

Kamlet, M.J., and Dickinson, C. (1968). Chemistry of detonations. III. Evaluation of the simplified calculational method for chapman-jouguet detonation pressures on the basis of available experimental information. J. Chem. Phys. *48*, 43-50.

Keshavarz, M.H. (2005). Simple procedure for determining heats of detonation. Thermochim. Acta *428*, 95-99.

Keshavarz, M.H. (2007). Detonation velocity of pure and mixed CHNO explosives at maximum nominal density. J. Hazard. Mater. *141*, 536-539.

Keshavarz, M.H. (2007). Quick estimation of heats of detonation of aromatic energetic compounds from structural parameters. J. Hazard. Mater. *143*, 549-554.

Keshavarz, M.H. (2008). Estimating heats of detonation and detonation velocities of aromatic energetic compounds. Propellants, Explos., Pyrotech. *33*, 448-453.

Keshavarz, M.H. (2012). A simple way to predict heats of detonation of energetic compounds only from their molecular structures. Propellants, Explos., Pyrotech. *37*, 93-99.

Keshavarz, M.H., and Pouretedal, H.R. (2005). Predicting the detonation velocity of CHNO explosives by a simple method. Propellants, Explos., Pyrotech. *30*, 105-108.

Kettner, M.A., Karaghiosoff, K., Klapötke, T.M., Sućeska, M., and Wunder, S. (2014). 3, 3′-Bi (1, 2, 4-oxadiazoles) Featuring the Fluorodinitromethyl and Trinitromethyl Groups. Chemistry–A European Journal *20*, 7622-7631.

Khire, V., Talawar, M., Prabhakaran, K., Mukundan, T., and Kurian, E. (2005). Spectro-thermal decomposition study of 1, 4-dinitroglycoluril (DINGU). J. Hazard. Mater. *119*, 63-68.

Kim, S., Chen, J., Cheng, T., Gindulyte, A., He, J., He, S., Li, Q., Shoemaker, B.A., Thiessen, P.A., Yu, B., et al. (2018). PubChem 2019 update: improved access to chemical data. Nucleic Acids Res. *47*, D1102-D1109.

Klapötke, T.M., Mieskes, F., Stierstorfer, J., and Weyrauther, M. (2016). Studies on Energetic Salts Based on (2, 4, 6-Trinitrophenyl) guanidine. Propellants, Explos., Pyrotech. *41*, 217-222.

Klapötke, T.M., Stierstorfer, J., Weyrauther, M., and Witkowski, T.G. (2016). Synthesis and Investigation of 2, 6-Bis (picrylamino)-3, 5-dinitro-pyridine (PYX) and Its Salts. Chemistry–A European Journal *22*, 8619-8626.

Klapötke, T.M., and Witkowski, T.G. (2016). 5, 5′-Bis (2, 4, 6-trinitrophenyl)-2, 2′-bi (1, 3, 4-oxadiazole)(TKX-55): Thermally Stable Explosive with Outstanding Properties. ChemPlusChem *81*, 357-360.

Kofler, A., and Brandstätter, M. (1948). Zur isomorphen vertretbarkeit von H, OH, Cl: S-trinitrobenzol, pikrinsäure, pikrylchlorid. Monatshefte für Chemie und verwandte Teile anderer Wissenschaften *78*, 65-70.

Kumar, D., Tang, Y., He, C., Imler, G.H., Parrish, D.A., and Shreeve, J.N.M. (2018). Multipurpose Energetic Materials by Shuffling Nitro Groups on a 3, 3′-Bipyrazole Moiety. Chemistry–A European Journal *24*, 17220-17224.

Kwasny, M., and Syczewski, M. (1980). Preparation and some physicochemical properties of compounds with trinitromethyl group. Biul. Wojsk. Akad. Tech. Im. Jaroslawa Dabrowskiego *29*, 165-172.

Lange, K., Koenig, A., Roegler, C., Seeling, A., and Lehmann, J. (2009). NO donors. Part 18: Bioactive metabolites of GTN and PETN—Synthesis and vasorelaxant properties. Bioorg. Med. Chem. Lett. *19*, 3141-3144.

Lee, J., Hsu, C., and Chang, C. (2002). A study on the thermal decomposition behaviors of PETN, RDX, HNS and HMX. Thermochim. Acta *392*, 173-176.

Lee, P.P., and Back, M.H. (1986). Kinetic studies of the thermal decomposition of tetryl using accelerating rate calorimetry: part I. Derivation of the activation energy for decomposition. Thermochim. Acta *107*, 1-16.

Leemann, H., and Grandmougin, E. (1908). Zur Kenntnis des symm. Hexanitro-azobenzols. Ber. Dtsch. Chem. Ges. *41*, 1295-1305.

Leonard, N.J., Miller, L.A., and Thomas, P.D. (1956). Unsaturated amines. VIII. Dehydrogenation and hydroxylation of 1-methyldecahydroquinoline by means of mercuric acetate1. J. Am. Chem. Soc. *78*, 3463-3468.

Leonard, P.W., Pollard, C.J., Chavez, D.E., Rice, B.M., and Parrish, D.A. (2011). 3, 6-Bis (4-nitro-1, 2, 5-oxadiazol-3-yl)-1, 4, 2, 5-dioxadiazene (BNDD): A Powerful Sensitive Explosive. Synlett *22*, 2097-2099.

Lewis, R.J., and Sax, N. (1996). Sax's dangerous properties of industrial materials (New York).

Li, B., Dong, H., and Zhang, J. (2003). Thermal Properties of Main By-products in TATB. Chin. J. Energ. Mater. *11*, 85-87.

Li, C., Liang, L., Wang, K., Bian, C., Zhang, J., and Zhou, Z. (2014). Polynitro-substituted bispyrazoles: a new family of high-performance energetic materials. J. Mater. Chem. A *2*, 18097-18105.

Li, H., An, C., Wen, X., Wang, J., and Du, M. (2016). Study on kinetic parameters of thermal decomposition reaction and thermal stability of 3, 4-bis (3-nitrofurazan-4-yl) furoxan based on kissinger method. Chin. J. Explos. Propellants *39*, 58-65.

Li, H., Li, D., Zeng, X., Liu, K., Beckers, H., Schaefer Iii, H.F., Esselman, B.J., and Mcmahon, R.J. (2015). Toward Understanding the Decomposition of Carbonyl Diazide (N3) 2CO and Formation of Diazirinone cycl-N2CO: Experiment and Computations. J. Phys. Chem. A *119*, 8903-8911.

Li, H., Zhang, L., Petrutik, N., Wang, K., Ma, Q., Shem-Tov, D., Zhao, F., and Gozin, M. (2019). Molecular and crystal features of thermostable energetic materials: guidelines for architecture of "bridged" compounds. ACS Cent. Sci. *6*, 54-75.

Li, W., Li, Z., and Wang, W. (2009). Preparation and Properties of TOATF. Chin. J. Energ. Mater. *17*, 11-13.

Licht, H., and Ritter, H. (1988). 2, 4, 6‐Trinitropyridine and Related Compounds, synthesis and characterization. Propellants, Explos., Pyrotech. *13*, 25-29.

Liu, L., Jin, X., Wang, P., Zhou, X., and Lu, M. (2016). Synthesis Improvement and Thermal Properties of Bis(2,2,2-Trinitroethyl)-Nitramine (BTNNA). Explos. Mater. *45*, 47-50.

Liu, L., Zhang, Y., Zhang, S., and Fei, T. (2015). Heterocyclic Energetic Salts of 4, 4′, 5, 5′-Tetranitro-2, 2′-Biimidazole. J. Energ. Mater. *33*, 202-214.

Liu, N., Shu, Y., Li, H., Zhai, L., Li, Y., and Wang, B. (2015). Synthesis, characterization and properties of heat-resistant explosive materials: polynitroaromatic substituted difurazano [3, 4-b: 3′, 4′-e] pyrazines. RSC Adv. *5*, 43780-43785.

Liu, N., Wang, B., Liu, H., Li, Y., Huo, H., Zhai, L., and Lai, W. (2015). Synthesis Crystal Structure and Thermal Properties of Two Furazano [3,4-b] tetrazolo [1,2-d] pyrazines. Chin. J. Energ. Mater. *23*, 13-17.

Makhova, N.N., Epishina, M.A., Ovchinnikov, I.V., and Pivina, T.S. (2003). New macrocyclic systems containing difurazanyl and furazanofuroxanyl units. Int. Annu. Conf. ICT *34th*, 80.

Manelis, G., Nazin, G., and Prokudin, V. (2006) Published. The additional activation volume of unimolecular reactions in the solid phase. Doklady Physical Chemistry, 2006 of Conference.: Springer, 335-338.

Mcneil, S.K., Kelley, S.P., Beg, C., Cook, H., Rogers, R.D., and Nikles, D.E. (2013). Cocrystals of 10-methylphenthiazine and 1, 3-dinitrobenzene: implications for the optical sensing of TNT-based explosives. ACS Appl. Mater. Interfaces *5*, 7647-7653.

Meyer, R., Köhler, J., and Homburg, A. (2016). Explosives (John Wiley & Sons).

Meyer, R., Köhler, J., and Homburg, D.I.A. (2007). Explosives (Wiley‐VCH Verlag GmbH & Co. KGaA).

Mo, Z., Zhang, A., Cao, X., Liu, Q., Xu, X., An, H., Pei, W., and Zhu, S. (2010). JASMIN: a parallel software infrastructure for scientific computing. Front. Comput. Sci. China *4*, 480–488.

Nair, U., Gore, G., Sivabalan, R., Pawar, S., Asthana, S., and Venugopalan, S. (2007). Preparation and

thermal studies on tetranitrodibenzo tetraazapentalene (TACOT): A thermally stable high explosive. J. Hazard. Mater. *143*, 500-505.

Nielsen, A.T., Atkins, R.L., and Norris, W.P. (1979). Oxidation of poly (nitro) anilines to poly (nitro) benzenes. Synthesis of hexanitrobenzene and pentanitrobenzene. J. Org. Chem. *44*, 1181-1182.

Ohta, A., Ogihara, Y., Nei, K., and Shibata, S. (1963). On Methylphenylnaphthalenes. I. Syntheses of Methylphenylnaphthalenes. Chem. Pharm. Bull. *11*, 754-758.

Pepekin, V., Matyushin, Y.N., and Gubina, T. (2011). Enthalpy of formation and explosive properties of 5, 6-(3, 4-furazano)-1, 2, 3, 4-tetrazine-1, 3-dioxide. Russ. J. Phys. Chem. B *5*, 97.

Politzer, P., and Murray, J.S. (2011). Some perspectives on estimating detonation properties of C, H, N, O compounds. Cent. Eur. J. Energ. Mater. *8*, 209-220.

Qu, Y., and Babailov, S.P. (2018). Azo-linked high-nitrogen energetic materials. J. Mater. Chem. A *6*, 1915-1940.

Rice, B.M., and Hare, J. (2002). Predicting heats of detonation using quantum mechanical calculations. Thermochim. Acta *384*, 377-391.

Rieckmann, T., Völker, S., Lichtblau, L., and Schirra, R. (2001). Investigation on the thermal stability of hexanitrostilbene by thermal analysis and multivariate regression. Chem. Eng. Sci. *56*, 1327-1335.

Roháč, M., Zeman, S., and RůŽičKa, A. (2008). Crystallography of 2, 2′, 4, 4′, 6, 6′-Hexanitro-1, 1′-biphenyl and Its Relation to Initiation Reactivity. Chem. Mater. *20*, 3105-3109.

Rothstein, L.R., and Petersen, R. (1979). Predicting high explosive detonation velocities from their composition and structure. Propellants, Explos., Pyrotech. *4*, 56-60.

Saraf, S., Rogers, W., and Mannan, M.S. (2003). Prediction of reactive hazards based on molecular structure. J. Hazard. Mater. *98*, 15-29.

Šarlauskas, J. (2010). Polynitrobenzenes containing alkoxy and alkylenedioxy groups: potential HEMs and precursors of new energetic materials. Cent. Eur. J. Energ. Mater. *7*, 313-324.

Schmidt, J., and Gehlen, H. (1965). PK-werte von derivaten des 1, 2, 4-triazols. Zeitschrift für Chemie *5*, 304-304.

Sheremetev, A.B., Kulagina, V.O., Aleksandrova, N.S., Dmitriev, D.E., Strelenko, Y.A., Lebedev, V.P., and Matyushin, Y.N. (1998). Dinitro trifurazans with oxy, azo, and azoxy bridges. Propellants, Explos., Pyrotech. *23*, 142-149.

Sheremetev, A.B., Kulagina, V.O., and Ivanova, E.A. (1996). Zero-hydrogen furazan macrocycles with oxy and azo bridges. J. Org. Chem. *61*, 1510-1511.

Sheremetev, A.B., Lyalin, B.V., Kozeev, A.M., Palysaeva, N.V., Struchkova, M.I., and Suponitsky, K.Y. (2015). A practical anodic oxidation of aminofurazans to azofurazans: an environmentally friendly route. RSC Adv. *5*, 37617-37625.

Siele, V., and Warman, M. (1962). Preparation of 1, 3-Difluoro-2, 4, 6-trinitrobenzene. J. Org. Chem. *27*, 1910-1911.

Sikder, N., Sikder, A., Bulakh, N., and Gandhe, B. (2004). 1, 3, 3-Trinitroazetidine (TNAZ), a melt-cast explosive: synthesis, characterization and thermal behaviour. J. Hazard. Mater. *113*, 35-43.

Sinditskii, V., Burzhava, A., Chernyi, A., Shmelev, D., Apalkova, V., Palysaeva, N., and Sheremetev, A. (2016). A comparative study of two difurazanyl ethers. J. Therm. Anal. Calorim. *123*, 1431-1438.

Singh, A., Sikder, N., and Sikder, A.K. (2005). Improved synthesis of an energetic material, 1, 3, 3-

trinitroazetidine (TNAZ) exploiting 2-iodoxy benzoic acid (IBX) as an oxidising agent. Indian J. Chem. *44B*, 2560-2563.
Spencer, E., and Wright, G.F. (1946). Preparation of picramide. Can. J. Res. *24*, 204-207.
Srinivasan, P., Gunasekaran, M., Kanagasekaran, T., Gopalakrishnan, R., and Ramasamy, P. (2006). 2, 4, 6-trinitrophenol (TNP): An organic material for nonlinear optical (NLO) applications. J. Cryst. Growth *289*, 639-646.
Tang, Y., He, C., Imler, G.H., Parrish, D.A., and Jean'ne, M.S. (2018). Ring closure of polynitroazoles via an N, N′-alkylene bridge: towards high thermally stable energetic compounds. J. Mater. Chem. A *6*, 8382-8387.
Tang, Y., He, C., Imler, G.H., Parrish, D.A., and Jean’ne, M.S. (2018). AC–C bonded 5, 6-fused bicyclic energetic molecule: exploring an advanced energetic compound with improved performance. Chem. Commun. *54*, 10566-10569.
Tang, Y., Kumar, D., and Shreeve, J.N.M. (2017). Balancing excellent performance and high thermal stability in a dinitropyrazole fused 1, 2, 3, 4-tetrazine. J. Am. Chem. Soc. *139*, 13684-13687.
Taylor, H.A., and Vesselovsky, V.V. (2002). The thermal decomposition of nitromethane. J. Phys. Chem. *39*, 1095-1102.
Terrier, F., Xie, H.Q., and Farrell, P.G. (1990). The effect of nitro-substitution upon diphenylmethane reactivity. J. Org. Chem. *55*, 2610-2616.
Tian, D., Zhao, F., and Liu, J. (2011). Handbook of energetic materials and the related compounds (National Defense Industry Press).
Trotter, J. (1960). The crystal structure of 1, 5-dinitronaphthalene. Acta Crystallogr. *13*, 95-99.
Tsyshevsky, R., Pagoria, P., Smirnov, A.S., and Kuklja, M.M. (2017). Comprehensive end-to-end design of novel high energy density materials: II. Computational modeling and predictions. J. Phys. Chem. C *121*, 23865-23874.
Türker, L. (2012). A trigonometric approach to a limiting law on detonation velocity. Match-Communications in Mathematical and Computer Chemistry *67*, 127.
Ulpiani, C. (1912). Constitution of Fulminuric Acids. IV. Compounds of the Formula H2(C2N2O3). Gazz. Chim. Ital. *42*, 243-63.
Veauthier, J.M., Chavez, D.E., Tappan, B.C., and Parrish, D.A. (2010). Synthesis and characterization of furazan energetics ADAAF and DOATF. J. Energ. Mater. *28*, 229-249.
Volk, F., and Bathelt, H. (1997). Influence of energetic materials on the energy-output of gun propellants. Propellants, Explos., Pyrotech. *22*, 120-124.
Wang, G., Xiao, H., Ju, X., and Gong, X. (2006). Calculation of detonation velocity, pressure, and electric sensitivity of nitro arenes based on quantum chemistry. Propellants, Explos., Pyrotech. *31*, 361-368.
Wang, G., Xiao, H., Xu, X., and Ju, X. (2006). Detonation velocities and pressures, and their relationships with electric spark sensitivities for nitramines. Propellants, Explos., Pyrotech. *31*, 102-109.
Wang, H., Wang, Y., Li, Y., Liu, Y., and Tan, Y. (2014). Scale-up synthesis and characterization of 2, 6-diamino-3, 5-dinitropyrazine-1-oxide. Def. Technol. *10*, 343-348.
Wang, Y., Yang, Z., Li, H., Zhou, X., Zhang, Q., Wang, J., and Liu, Y. (2014). A novel cocrystal explosive of HNIW with good comprehensive properties. Propellants, Explos., Pyrotech. *39*, 590-596.
Wei, J., Li, F., Xu, J., and Peng, X. (2015). Synthesis and thermal stability of new polynitrostilbenes.

Aust. J. Chem. *68*, 919-925.

Wikipedia. 2006. Kamlet-Jacobs-Gleichungen [Online]. Available: https://de.wikipedia.org/wiki/Kamlet-Jacobs-Gleichungen.

Wu, J., Zhang, J., Li, T., Li, Z., and Zhang, T. (2015). A novel cocrystal explosive NTO/TZTN with good comprehensive properties. RSC Adv. *5*, 28354-28359.

Wurzenberger, M.H., Lechner, J.T., Lommel, M., Klapötke, T.M., and Stierstorfer, J. (2020). Salts of Picramic Acid–Nearly Forgotten Temperature‐Resistant Energetic Materials. Propellants, Explos., Pyrotech.

Yan, T., Cheng, G., and Yang, H. (2019). 1, 2, 4‐Oxadiazole‐Bridged Polynitropyrazole Energetic Materials with Enhanced Thermal Stability and Low Sensitivity. ChemPlusChem *84*, 1567-1577.

Yang, C.H., Lu, Y.M., Yan, M.Q., Li, J., Wu, J., Li, Q.Y., Yang, J., Shen, L., Yang, G.W., and Zou, J.H. (2016). Nitrogen–rich 1, 2‐bis (tetrazol‐5‐yl) ethane and its Carboxylate Derivative for Potential Energetic Materials. ChemistrySelect *1*, 2757-2761.

Yang, Z., Li, H., Zhou, X., Zhang, C., Huang, H., Li, J., and Nie, F. (2012). Characterization and properties of a novel energetic–energetic cocrystal explosive composed of HNIW and BTF. Cryst. Growth Des. *12*, 5155-5158.

Yang, Z., Zeng, Q., Zhou, X., Zhang, Q., Nie, F., Huang, H., and Li, H. (2014). Cocrystal explosive hydrate of a powerful explosive, HNIW, with enhanced safety. RSC Adv. *4*, 65121-65126.

Yin, P., and Shreeve, J.N.M. (2015). From N‐Nitro to N‐Nitroamino: Preparation of High‐Performance Energetic Materials by Introducing Nitrogen‐Containing Ions. Angew. Chem. *127*, 14721-14725.

Yin, P., Zhang, J., Imler, G.H., Parrish, D.A., and Shreeve, J.N.M. (2017). Polynitro‐Functionalized Dipyrazolo‐1, 3, 5‐triazinanes: Energetic Polycyclization toward High Density and Excellent Molecular Stability. Angew. Chem. *129*, 8960-8964.

Yin, P., Zhang, J., Parrish, D.A., and Jean'ne, M.S. (2014). Energetic N, N′‐Ethylene‐Bridged Bis (nitropyrazoles): Diversified Functionalities and Properties. Chemistry–A European Journal *20*, 16529-16536.

Zaitsev, A., Kortusov, I., Dalinger, I., Kachala, V., Popova, G., and Shevelev, S. (2009). Nitropyrazoles 16. The use of methoxymethyl group as a protecting group for the synthesis of 4-methyl-3-nitro-5-R-pyrazoles. Russ. Chem. Bull. *58*, 2118-2121.

Zeman, S. (1980). Possibilities of applying Piloyan method of determination of decomposition activation energies in differential thermal analysis of polynitroaromatic compounds and their derivatives: Part IV. 1, 3, 5-trinitrobenzene, 2, 2′, 4, 4′, 6, 6′-hexanitrobiphenyl, 2, 2′, 2 ″, 4, 4′, 4 ″, 6, 6′, 6 ″-nonanitro-m-terphenyl, 1, 4, 5, 8-tetranitronaphthalene and 2, 4, 6-tripicryl-l, 3, 5-triazine. J. Therm. Anal. Calorim. *19*, 207-214.

Zeman, S. (1993). The thermoanalytical study of some aminoderivatives of 1, 3, 5-trinitrobenzene. Thermochim. Acta *216*, 157-168.

Zeman, S. (2003). New aspects of impact reactivity of polynitro compounds. Part IV. Allocation of polynitro compounds on the basis of their impact sensitivities. Propellants, Explos., Pyrotech. *28*, 308-313.

Zeman, S., Roháč, M., Friedl, Z., Růžička, A., and Lyčka, A. (2010). Crystallography and Structure–Property Relationships of 2, 2 ″, 4, 4′, 4 ″, 6, 6′, 6 ″‐Octanitro‐1, 1′: 3′, 1 ″‐Terphenyl (ONT).

Propellants, Explos., Pyrotech. *35*, 130-135.
Zeng, Z., Guo, Y., Twamley, B., and Jean'ne, M.S. (2009). Energetic polyazole polynitrobenzenes and their coordination complexes. Chem. Commun., 6014-6016.
Zhang, H., Guo, C., Wang, X., Xu, J., He, X., Liu, Y., Liu, X., Huang, H., and Sun, J. (2013). Five energetic cocrystals of BTF by intermolecular hydrogen bond and π-stacking interactions. Cryst. Growth Des. *13*, 679-687.
Zhang, J., Wang, J., Xu, H., and Zhou, X. (2013). Synthesis and thermal decomposition kinetics of hexanitroazobenzene. Chin. J. Energ. Mater. *21*, 7-11.
Zhang, J., and Xiao, H. (2002). Computational studies on the infrared vibrational spectra, thermodynamic properties, detonation properties, and pyrolysis mechanism of octanitrocubane. J. Chem. Phys. *116*, 10674-10683.
Zhang, J., Xue, B., Rao, G., Chen, L., and Chen, W. (2018). Thermal decomposition characteristic and kinetics of DINA. J. Therm. Anal. Calorim. *133*, 727-735.
Zhang, L., Jiang, S.-L., Yu, Y., Long, Y., Zhao, H.-Y., Peng, L.-J., and Chen, J. (2016). Phase Transition in Octahydro-1,3,5,7-tetranitro-1,3,5,7-tetrazocine (HMX) under Static Compression: An Application of the First-Principles Method Specialized for CHNO Solid Explosives. J. Phys. Chem. B *120*, 11510-11522.
Zhang, P., Kumar, D., Zhang, L., Shem-Tov, D., Petrutik, N., Chinnam, A.K., Yao, C., Pang, S., and Gozin, M. (2019). Energetic Butterfly: Heat-Resistant Diaminodinitro trans-Bimane. Molecules *24*, 4324.
Zhang, X., Dong, H., Zhou, Z., and Li, H. (2010). Synthesis and Properties of 3-Amino-2,4,6-Trinitrophenol and 3,5-Diamino-2,4,6-Trinitrophenol. Acta Armamentarii *31*, 1341-1345.
Zhang, X., Xiong, H., Yang, H., and Cheng, G. (2017). 1 4, 1 6, 3 4, 3 6, 5 4, 5 6, 7 4, 7 6-Octanitro-2, 4, 6, 8-tetraoxa-1, 3, 5, 7 (1, 3)-tetrabenzenacyclooctaphane and its derivatives: thermally stable explosives with outstanding properties. New J. Chem. *41*, 5764-5769.
Zhang, Y., Zhou, C., Wang, B., Zhou, Y., Xu, K., Jia, S., and Zhao, F. (2014). Synthesis and Characteristics of Bis (nitrofurazano) furazan (BNFF), an Insensitive Material with High Energy-Density. Propellants, Explos., Pyrotech. *39*, 809-814.
Zhao, X., and Liu, Z. (2013). 2, 6-Diamino-3, 5-dinitropyrazine-1-oxide synthesis and its explosion properties. J. Chem. Eng. Chin. Univ. *2*, 248.
Zhou, Y., Wang, B., Li, J., Zhou, C., Hu, L., Chen, Z., and Zhang, Z. (2011). Study on Synthesis, Characterization and Properties of 3,4-Bis(4'-nitrofurazano-3'-yl)furoxan. Acta Chim. Sin. (Chin. Ed.) *69*, 1673-1680.
